# The Challenge of Analyzing the Sugarcane Genome

**DOI:** 10.3389/fpls.2018.00616

**Published:** 2018-05-14

**Authors:** Prathima P. Thirugnanasambandam, Nam V. Hoang, Robert J. Henry

**Affiliations:** ^1^Queensland Alliance for Agriculture and Food Innovation, The University of Queensland, St. Lucia, QLD, Australia; ^2^ICAR - Sugarcane Breeding Institute, Coimbatore, India; ^3^College of Agriculture and Forestry, Hue University, Hue, Vietnam

**Keywords:** sugarcane genome, genome sequencing, genome translating, polyploid genome, sugarcane sequencing, progenitors species, comparative genomics

## Abstract

Reference genome sequences have become key platforms for genetics and breeding of the major crop species. Sugarcane is probably the largest crop produced in the world (in weight of crop harvested) but lacks a reference genome sequence. Sugarcane has one of the most complex genomes in crop plants due to the extreme level of polyploidy. The genome of modern sugarcane hybrids includes sub-genomes from two progenitors *Saccharum officinarum* and *S. spontaneum* with some chromosomes resulting from recombination between these sub-genomes. Advancing DNA sequencing technologies and strategies for genome assembly are making the sugarcane genome more tractable. Advances in long read sequencing have allowed the generation of a more complete set of sugarcane gene transcripts. This is supporting transcript profiling in genetic research. The progenitor genomes are being sequenced. A monoploid coverage of the hybrid genome has been obtained by sequencing BAC clones that cover the gene space of the closely related sorghum genome. The complete polyploid genome is now being sequenced and assembled. The emerging genome will allow comparison of related genomes and increase understanding of the functioning of this polyploidy system. Sugarcane breeding for traditional sugar and new energy and biomaterial uses will be enhanced by the availability of these genomic resources.

## Introduction

*“Amongst the sugarcane we are safe”* So, says the Chinese with sugarcane (*Saccharum* spp.) symbolizing bravery, independence and protection (DeBernardi, 2009). Sugarcane stalks also signified the power of divine protection in many traditions, including Indian. The name sugarcane is used to refer to a group of tall perennial tropical grass species which were domesticated for sugar production, and have been classified inconsistently (Paterson et al., 2013). Sugarcane has been known for more than 2,200 years and it was one of the first plants to inspire humans to develop technology (Goldstein and Mintz, 2015). The earliest known crystal sugar production was in India, wherein crushing and boiling the sugarcane juice was practiced (Gopal, 1964). Since then, the sugar production processes have not changed much in principle. To date, sugarcane production by weight exceeds that of any of the food crops such as wheat, rice or maize (FAO, 2017).

Sugarcane was one of the earliest inspirations for use of technology by humans, but the crop has been given less attention in the area of scientific research. Major discoveries such as C4 photosynthesis (Hatch et al., 1967; Hatch, 2005) were made in sugarcane, however, many of the related physiological and biochemical processes remain unexplored. Despite being the highest accumulator of sucrose, the crop has not been studied in detail. One of the reason being a tropical crop, the majority of sugarcane research is pioneered mainly by countries like Brazil and Australia, unlike maize and wheat that garners worldwide attention. Another reason is the polyploid and heterozygous nature of its genome. For decades, sugarcane genomics has lagged behind than that for other grass species including rice, wheat, barley, and sorghum due to its very complex and polyploid genome. However, recently, sugarcane has become the foremost candidate crop for bioenergy and biomaterial production as a replacement for oil and has attracted research interest globally as energy demand surges and the quest for sustainable options increases (for reviews, see de Souza et al., 2014; Hoang et al., 2015a). Consequently, the sugarcane genome is a focal point that holds the answers to many intriguing aspects of sugarcane.

The genetics of sugarcane is now known to be one of the most complex that exists in the plant kingdom. The very complex genome that was a barrier to analysis and sequencing has now attracted the scientific community. Novel plant breeding approaches are required to mitigate some of the worst scenarios of climate change and ensure sustainable sugarcane production. The recent genomic advances help breeders by providing them with a great opportunity to incorporate the diversity of alleles into the breeding programs, through gene mining from wild relatives (Abberton et al., 2016). The Diversity Seek initiative consortium^1^ was launched in 2015 aiming to provide data on diverse germplasm and facilitate the characterization of germplasm and application of genomics tools to identification of rare novel useful alleles for incorporation into current germplasm. In the genome-based era of crop improvement, this initiative will help safeguard our future through increased food security (Abberton et al., 2016). Most of the technical difficulties associated with sugar and bioenergy/biomaterial production from sugarcane can be addressed by genetic approaches (Hotta et al., 2010; Furtado et al., 2014), for instance, using genomic assisted breeding programs will allow for input responsive genotype development (Scortecci et al., 2012), and understanding the sub-genomic origins of the important traits like fiber or sugar will help in designing breeding strategies for the end product specific sugarcane genotypes.

With advances in genomic tools and next generation sequencing methods, studies are beginning to unravel the nature of the complexity of the genome of sugarcane step by step and sugarcane could soon become a model for studying other complex, polyploid genomes. In recent years, sugarcane genomics has improved although not yet to the extent that has been achieved in other crops, such as the cereals. Our understanding of the evolutionary aspects and genome structure of sugarcane has improved thanks to significant resources such as genetic maps, large scale EST collections, transcriptomes, bacterial artificial chromosome (BAC) libraries and shotgun genome sequences (Souza et al., 2011). Sorghum has been widely accepted as a close diploid reference and the availability of efficient genetic transformation of sugarcane has resulted in several transgenics for different traits in the pipeline (Grivet and Arruda, 2002).

With this background, we outline the developments in characterizing the sugarcane genome and sub-genome structures, sequencing strategies and comparative genomics to provide a review of progress made in recent years in meeting the challenges of describing and translating the complex sugarcane genome.

## The Sugarcane Nuclear Sub-Genomes and Organellar Genomes

**FIGURE 1:**
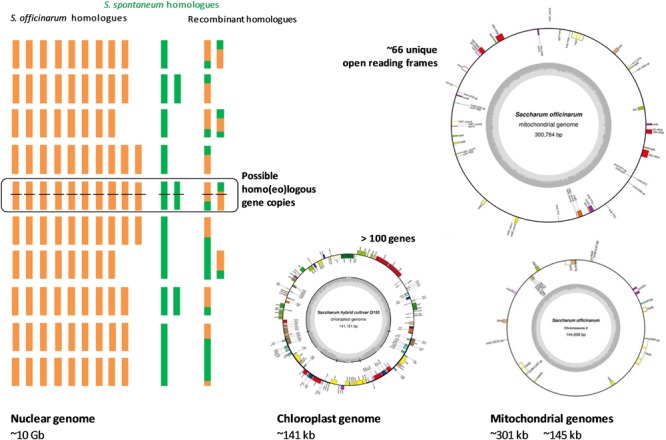
Scheme of the sugarcane hybrid nuclear sub-genomes and the genetic contributions of each parental species, the chloroplast genome and the mitochondrial genomes. Adapted from (D’Hont et al., 1996, 1998; Piperidis et al., 2010; Hoang et al., 2015b; Aitken et al., 2016; Shearman et al., 2016; Garsmeur et al., 2017).

The complex and large polyploid nuclear genome and organellar genomes of sugarcane pose great challenges to genome sequencing and contribute to the fact that sugarcane genomics has lagged behind, in comparison with other grass species such as rice, maize, and sorghum. Despite this, progress in unraveling the sugarcane nuclear and organellar genomes has been made in recent years due to the advances in sequencing technologies, available resources and the availability of the genome sequences of related species. The sugarcane genome structure including nuclear sub-genomes, their origins, chloroplast and mitochondrial genomes are illustrated in **Figure 1**.

### Sugarcane Nuclear Sub-Genomes

The nuclear genome of modern sugarcane (*Saccharum* spp. hybrids) is composed of sub-genomes originally from two species, a female thick-stalked, high-sugar *S. officinarum* and a wild male thin-stalked, low-sugar *S. spontaneum* (Daniels and Roach, 1987; D’Hont and Glaszmann, 2001). *S. officinarum* is an octoploid species, which has a basic chromosome number (*x*) of 10, a basic monoploid genome size of ∼1 Gb, and a total number of chromosomes (2*n*) of 80 (D’Hont et al., 1996, 1998; Zhang et al., 2012). This results in a total genome size of about ∼ 7.88 Gb for this species (Zhang et al., 2012). The genome is autopolyploid, which means that there are eight homologous copies of each gene in the genome of the *S. officinarum*. *S. spontaneum*, on the other hand, has a basic chromosome number of 8, basic monoploid genome size of 750–843 Mb, varied ploidy levels with the total chromosome number ranging from 40 to 128 and a genome size range reported to be from 3.36 to 12.64 Gb (Panje and Babu, 1960; Daniels and Roach, 1987; Sreenivasan et al., 1987; da Silva et al., 1995; D’Hont et al., 1998; Ha et al., 1999; Zhang et al., 2012). The ploidy level of this species can be between 5X and 16X, which suggests that this autopolyploid species has 5–16 homologous copies of each gene in its genome. The most frequently observed ploidy level for *S. spontaneum* is eight (Irvine, 1999). There are hybridization programs that involved other *Saccharum* species like *S. barberi* and *S. sinense* mainly contributing to the increased vigor, hardiness, tillering, disease resistance and environmental adaptations. *S. barberi* and *S. sinensis* are reported to have been derived from *S. officinarum* and *S. spontaneum* (Amalraj and Balasundaram, 2006) and *S. spontaneum* itself was likely to be involved in the origin of *S. officinarum* (Babu et al., 2010). The interaction of different genomes in a hybrid background and their contribution towards hybridity remain unclear, particularly because of their high and variable ploidy levels.

The interspecific hybrid genome of sugarcane possesses genetic materials inherited from both parental species unevenly, which makes the genome more complex than that of its progenitors (D’Hont et al., 1996). The hybrid genome has a basic chromosome number of 10 (similar to that of sorghum and maize), however, its complexity resides in the mixture of aneuploid and homo(eo)logous chromosomes, which results in the sugarcane genome having 10 uneven homo(eo)logous chromosome groups (Grivet et al., 1996). The total number of chromosomes in sugarcane has been reported to be between 100 and 130 (Simmonds, 1976; Sreenivasan et al., 1987), and it is estimated that there are 8–14 homo(eo)logous copies of a given gene at a given locus in the sugarcane genome (Heinz, 1987; Grivet and Arruda, 2002; Rossi et al., 2003; Aitken et al., 2004, 2016; Souza et al., 2011). The total number of chromosomes in the sugarcane genome differs from genotype to genotype (or cross to cross), due to the random sorting of the chromosomes in the genome in each crossing. For instance, a total of 110 chromosomes was reported for cultivar Q117, 107 chromosomes for cultivar Q200 (Piperidis et al., 2010; Aitken et al., 2016); 115 and 124 for Co281 and Co453, respectively; 108 and 103 for B49119 and B62163, respectively (Heinz, 1987). If only the monoploid (haploid) genome is considered, it is estimated that the sugarcane genome is about 1 Gb in size. However, the total actual size of the sugarcane nuclear genome is about 10 Gb (D’Hont and Glaszmann, 2001; Le Cunff et al., 2008), which is about three times larger than the human genome, about 10 times larger than the closest related species sorghum and about 20 times larger than rice genome (Aitken et al., 2016). The unequal contribution of each progenitor to the hybrid cultivar R570 genome was revealed by genomic *in situ* hybridization (GISH) and fluorescent *in situ* hybridization (FISH), demonstrating that the female parent *S. officinarum* contributed about 80% of the chromosomes to the genome of the hybrids, while the male parent *S. spontaneum* contributed only 10–20% to the hybrid genome (D’Hont et al., 1996; Piperidis et al., 2001; Cuadrado et al., 2004; D’Hont, 2005). It was also shown that the other chromosomes (5–17%) resulted from recombination of chromosomes from the two-parental species. Therefore, sugarcane hybrids are highly heterozygous, typically, possessing more than eight copies of homologous chromosomes originating from *S. officinarum*, a few copies (1–2) each of homologous chromosomes from *S. spontaneum* and interspecific recombinant chromosomes (Ming et al., 1998). It is important to note that, genetically, the uniqueness of each sugarcane hybrid cross most likely directly reflects the chromosome ratio originally from the two parental species, while phenotypically, the more the contribution of the wild *S. spontaneum*, the greater the fiber content, hardiness and all complementary characteristics in the hybrid (Matsuoka et al., 2014).

The mixture of homo(eo)logous chromosome sets from two polyploid progenitor species, together with a high repeat content (Figueira et al., 2012; Berkman et al., 2014), has impeded understanding of how the genome functions and construction of a reference genome for sugarcane (Souza et al., 2011; Figueira et al., 2012). It is worth mentioning that, despite the fact that there are no diploid progenitors of sugarcane, the challenges posed by the hybrid genome might mean that availability of genome sequences from the two autopolyploid progenitors could simplify the unraveling of the hybrid genome and provide more insights into the process of establishing the sequencing of the hybrid genome.

### Sugarcane Chloroplast Genome

The sugarcane chloroplast genome has been sequenced and assembled by different technologies, for different cultivars, including NCo310 - GenBank accession: AP006714.1 (Asano et al., 2004), SP80-3280 – GenBank accession: AE009947.2 (Calsa Junior et al., 2004), Q155 – GenBank accession: KU214867.1 (Hoang et al., 2015b) and RB867515 – GenBank accession: KX507245.1 (Vidigal et al., 2016). The length of the chloroplast genome reported for the two former cultivars is 141,182 bp, whereas for the two latter sequences is one base pair shorter. The chloroplast genomes obtained from cultivar Q155 (an Australian cultivar) and cultivar RB867515 (a Brazilian cultivar) are identical, and differing from the NCo310 (released in South Africa) by five polymorphisms (4 SNPs and 1 indel) and from the SP80-3280 (another Brazilian cultivar) by eight polymorphisms (6 SNPs and 2 indels) (Hoang et al., 2015b; Vidigal et al., 2016). The two chloroplast sequences of Q155 and RB867515 were based upon deep sequencing, being 2,357X and 7,675X, respectively, while that of SP80-3280 and NCo310 were derived from relatively low coverage (8X) shotgun sequencing and sequencing of PCR amplified fragments of the chloroplast genome, respectively. As shown in Hoang et al. (2015b) and Vidigal et al. (2016), the discrepancies between these two groups of chloroplast genomes include SNPs within genes *atp*A, *psb*C, *rrn*23, *trn*G, *trn*M, and *trn*S; and in the intergenic regions of the chloroplast genome. This could be due to errors in the early studies resulting from cross contamination by the chloroplast homologs inserted in the mitochondrial genome (which was recently revealed in the sugarcane mitochondrial genomes by Shearman et al. (2016), and introduced into the assembly of chloroplast genome at a low coverage or in the PCR amplification process (Hoang et al., 2015b). Nevertheless, the information obtained from these studies confirms highly conserved chloroplast genome sequences amongst the tested cultivars, as a result of the narrow genetic base of the sugarcane hybrids.

Low coverage of the chloroplast genomes from sugarcane progenitor species were also assembled by Berkman et al. (2014) and Evans and Joshi (2016) with a varied genome size and coverage. For instance, the *S. officinarum* cultivar IJ76-514 has a chloroplast genome of 141,176 bp with an average coverage of 34.95, while *S. spontaneum* SES234B has a chloroplast genome of 141,185 bp with an average coverage of 55.62. These genomes were assembled based on the chloroplast genome of the cultivar NCo310.

Up to 135 genes have been functionally annotated for the sugarcane chloroplast genome, including protein-coding genes, ribosomal RNA genes, and transfer RNA genes. Many of these genes were annotated in both inverted repeat regions of the chloroplast genome, including eight protein-coding genes (*ndh, rpl*2, *rpl*23, *ycf*2, *ycf*15, *ycf*68, *rps*7, *rps*15, and *rps*19), four ribosomal RNA genes (*rrn*4.5, *rrn*5, *rrn*16, and *rrn*23), and eight transfer RNA genes (*trn*A, *trn*H, 2 *trn*I, *trn*L, *trn*N, *trn*R, and *trn*V) (Vidigal et al., 2016). Comparative analysis from Asano et al. (2004) and Calsa Junior et al. (2004) suggested that the sugarcane chloroplast genome was more closely related to the maize and sorghum chloroplast genomes than to that of rice or wheat, indicating a common ancestor for the three former plastomes.

### Sugarcane Mitochondrial Genome

The sugarcane mitochondrial genome has been a challenge due to its complexity and high repetitive content. Several unsuccessful attempts have been made to obtain the genome from whole genome shotgun sequencing read data. In a recent study, using PacBio long-read technology, the mitochondrial genome of a commercial sugarcane hybrid, Khon Kaen 3, was captured in two discrete DNA circles (chromosomes) without evidence of recombination, and with all repeats in the genome covered by individual reads (Shearman et al., 2016). One larger mitochondrial chromosome is 300,778 bp in length (mean read depth was 13, GenBank accession: LC107874.1), while a smaller chromosome is 144,698 bp in length (mean read depth was 14, GenBank accession: LC107875.1). A total of 66 unique open reading frames, 26 duplicate sequences and 17 partial chloroplast homologous gene fragments inserted in the mitochondrial genome were annotated in the sugarcane mitochondrial genome. The authors reported no structural rearrangements between mitochondrial genomes of the hybrids and its progenitors, whereas, significant rearrangements between sugarcane and sorghum mitochondrial genomes were observed. Based on the large number of sequences linking the two chromosomes, the authors postulated that the separation of the two mitochondrial chromosomes could have occurred relatively recently.

## Sugarcane Genome Sequencing

### The Complications and Challenges in Sugarcane Genome Sequencing

The application of genomics and the next generation sequencing technologies in sugarcane faces imminent challenges some of which are discussed below.

### Homo(eo)logs and Gene Copy Number

In general, it has been suggested that one of the two genomes in an interspecific cross is preferentially retained in a merger (during the diploidization process), often with higher gene expression levels referred to as biased fractionation, and this has been reported for many angiosperm species (Wendel, 2015). Biased fractionation is observed in sugarcane where more genes are shown as being lost from the progenitor *S. spontaneum* than from *S. officinarum. In situ* hybridization using species-specific DNA probes from *S. officinarum* and *S. spontaneum* revealed that sugarcane hybrids are poly-aneuploids with recombination occurring between homoeologous chromosomes (D’Hont et al., 1996). This kind of genetic composition is very complex, reflecting the inherent polygenic control of the traits in sugarcane. Any gene locus and its allelic complement is represented multiple times as the homologous chromosomes occur in large number from each of the two sub genomes present in any hybrid. The number of alleles is reported to vary from 8 to 14 (Rossi et al., 2003; Aitken et al., 2004, 2014) as a result of which higher ploidy, polysomic pairing and reduction of single copy genes are observed. When the BAC clones of two homo(eo)logous sequences (97 and 126 kb) of *Adh*1 gene were analyzed in the sugarcane hybrid R570, a high collinearity and gene structure conservation were observed between the two homo(eo)logous haplotypes (Jannoo et al., 2007). A high homology was also observed in the heterochromatin region except for a few insertions of retrotransposable elements. This study revealed that there is not much genetic remodeling of the merged genomes due to the high ploidy levels which is usually reported to cause generalized rearrangement of genomes (Jannoo et al., 2007). In another study, BAC clones belonging to seven homo(eo)logous haplotypes were sequenced, for comparing homoeologous and also homologous haplotypes from *S. spontaneum* or *S. officinarum* (Garsmeur et al., 2011). The sequence identity was studied for each pair of homo(eo)logous and orthologous genes. A high sequence identity with an average of 95.9% was reported in the coding regions and an average of 87.5% in the intronic regions of the homo(eo)logs was reported. Studies to quantify homologous chromosomes in sugarcane reported non-additive allele dosage, with one or two copies having favorable effects, while the other copies have negative effects (Ming et al., 2001). Sugarcane and sorghum showed an average identity of 91.6% in the coding regions and 72.8% in the non-coding regions, while sugarcane and rice had a lower average identity, with 71% for the coding regions and 38% for the non-coding regions (Garsmeur et al., 2011). The consequences of a merger of two diverged genomes have been studied in several polyploid plants including *Tragopogan, Glycine, Arabidopsis, Triticum, Brassica, Nicotiana*, and *Oryza* (Wendel, 2015). At the genomic level, modifications in the genome include mutagenic gene silencing or deletions, intergenomic transfer of repetitive elements, differential rates of accumulation of nucleotide substitutions, and various forms of homoeologue interaction resulting in chimeras or duplicated genes. These phenomena are the general outcome of polyploidization though they might vary in nature and extent among the polyploid systems and in most cases their phenotypic or ecological or evolutionary consequences are not known (Wendel, 2015). At the functional level, a variety of short-term evolutionary responses to polyploidy occurs, including non-Mendelian epigenomic and regulatory RNA alterations, reprogramming of the transcriptome, proteome and metabolome affecting plant phenotype and function that might provide higher functional plasticity (Jackson and Chen, 2010; Song and Chen, 2015). The merger of two different genomes which themselves are polyploids, in the case of sugarcane has to be studied in detail and no information on interactions and the subsequent alterations and modifications of the genetic material is available yet. In sugarcane, developing an understanding of these myriad genetic interactions and their evolutionary consequences is an exciting area for investigation in the coming years, with new advances and technical robustness applied to this unique polyploid system.

### Whole Genome Duplications

Polyploidy in plants is wide-spread and all plant species have undergone at least one round of whole genome duplication (WGD) in their evolutionary pathway, and at least 15% of speciation events are thought to be associated with increased ploidy (Grandont et al., 2013). WGD has a very common occurrence in plants wherein the entire genome is duplicated. It is established as an important evolutionary tool of plant speciation and crop domestication. After polyploidization, rapid reorganization of the genomic structure occurs. A WGD event was reported to have occurred in the *Saccharum* lineage, after it diverged from the sorghum lineage about 10 MYA (Paterson et al., 2012). The Saccharinae group of grasses is said to be an intriguing system for exploring recent genome duplications in a genome and its widespread effect on evolutionary processes (Kim et al., 2014). The merging of the sub-genomes (or heteromes) and “maintenance of duplicate genes” (Paterson et al., 2004) in a hybrid have been studied for a long time. A pan-cereal WGD event (also known as *rho*) was reported to have occurred about 65–70 MYA, earlier than the divergence between the PACMAD (Panicoideae, Arundinoideae, Chloridoideae, Micrairoideae, Aristidoideae, and Danthonioideae) and the BEP (Bambusoideae, Ehrhartoideae, and Pooideae) clades (Paterson et al., 2004; Wang et al., 2005) after which the *Saccharum* genome is said to have undergone two WGD events, while the sorghum genome has not undergone any additional genome duplication (Kim et al., 2013) (discussed later). Though sorghum and sugarcane are reported to have high genetic similarity, in a study only 6.4% of BAC clones could be anchored to the sorghum genome which might have been due to the genomic rearrangements in the *Saccharum* genus as a result of two WGD events after its divergence from sorghum (Kim et al., 2013). The occurrence of two additional genome duplication events is further known from *Saccharum* having 2n = 80, with a homologue dosage of about eight while the ancestral progenitor must have been similar to the modern sorghum with a chromosome number of 10 (Kim et al., 2014). Although *Saccharum* has undergone two WGD events, it shares extensive collinearity and low genomic rearrangements with maize, rice and *Brachypodium* (Ming et al., 1998). With the available and on-going genome sequencing of many grass species especially sorghum, maize, rice, *Setaria* and *Brachypodium*, comparative genomic research for sugarcane is made much easier with sorghum as a reference genome for sugarcane. However, sugarcane lacks finer studies to answer a number of unresolved questions related to whole genome doubling (WGD) and duplicate maintenance in the polyploid hybrid.

### Transposons and Repetitive Content

Transposable elements (TE) are capable of causing many kinds of genetic variation, in the course of plant evolution. TEs represent an endogenous system that provides a degree of evolvability that would not be available otherwise in genomes. TEs played a major role in the trajectory of plant evolution and adaptation (Lisch, 2013). TEs are capable of “generating genomic plasticity” by introducing mutations and thereby creating allelic diversity (Lee and Kim, 2014). It is reported that repetitive content among the published plant genomes are found to vary from 3% (in bladderwort) to 85% (in maize) with an average repetitive content of 46% per genome (Abdurakhmonov, 2016). There is some direct correlation between genome size and repetitive content, with some exceptions. Norway spruce which has one of the largest genomes (19,600 Mb) and bladder wort, that has one of the smallest genome (77 Mb) were both found to have ∼28,000 genes, (Abdurakhmonov, 2016). In a study by Garsmeur et al. (2011), sugarcane BAC clones belonging to seven homo(eo)logous haplotypes of the rust resistance gene *Bru*1 and corresponding sorghum BAC clones were selected. It was found that 66 large TEs covered an average of 35% of all BAC sequences and about 21% of them were not reported earlier (Garsmeur et al., 2011). In addition, LTR retrotransposons were reported to be the most frequent TE elements, representing 65% and belonging to two superfamilies, namely Ty3-Gypsy and Ty1-Copia. Non-LTR retrotransposons represented 35% and mainly consisted of the LINE superfamily, DNA transposons and CACTA superfamily. Twenty percent of the TEs were found to be complete, including 12 LTR retrotransposons and one transposon. When the insertion times were calculated for all complete LTR retrotransposons, it was estimated to be in the range from 0 to 1.58 MYA. The majority of TEs were found to be located in intergenic regions, with no collinearity of their positions across haplotypes (Garsmeur et al., 2011).

It has been reported that almost one-half of the sugarcane BAC sequences are composed of TEs based on BAC-end sequencing studies from two sugarcane cultivars, R570 (42.8%) (Kim et al., 2013) and SP80-3280 (45.16%) (Figueira et al., 2012). This is likely to be an underestimation of the TE content in sugarcane as the BACs were mainly from euchromatic gene-rich regions (de Setta et al., 2014). In a study by Jannoo et al. (2007), a very high global homology was observed between the two homoeologous BAC clones of gene rich regions of *S. officinarum* and *S. spontaneum.* The study also states that the major difference between the two BAC clones could be mainly due to the TE content, which forms the basis for differential labeling studies to differentiate between the two genomes and that the differential labeling may be due to the qualitative and/or quantitative differences in TE content specific to each species. The repeat content of rice, sorghum, and maize genomes was reported to be 35% (International Rice Genome Sequencing Project, 2005), ∼61% (Paterson et al., 2009), and 85% (Schnable et al., 2009), respectively. The composition and occurrence of repeat sequences in sugarcane could be completely different to that in sorghum and maize despite belonging to the same tribe. In particular, when LTR retrotransposons were compared among sugarcane, sorghum and maize, Ty1-copia was found to be more abundant in sugarcane than Ty3-gypsy which is more abundant in maize and sorghum (de Setta et al., 2014). From the same study, it was also suggested that overall the sugarcane genome has undergone or is undergoing expansion compared with sorghum and about one-fourth of the expansion is due to the differences in TE content. In the genome sequencing context, the repeat content can be overcome by some of the methods described in Claros et al. (2012) which are as follows: (1) increasing the read length or a combination of short and long reads, (2) producing paired-end reads longer than the repeated regions, and (3) correlating contigs with genetic maps and/or FISH. As the sequencing platforms become improved to generate error-free reads with high coverage and assembly, the problems with repeat content of the genomes should be resolved. However, the recent advances in the sequencing technologies, such as single-molecule sequencing are giving longer reads (discussed later), which will clearly help in the resolution of long repetitive DNAs (Claros et al., 2012).

### Challenges in Sugarcane Genome Sequencing

It is well-known that, plant genomes are more complex, compared to other eukaryotic systems which creates challenges in the study of these genomes. Beginning from the isolation of high-quality DNA from plant tissues devoid of phenolic and other metabolic compounds and efficient library preparation for whole-genome sequencing, the processes are challenging. Sugarcane high molecular weight DNA extraction has been well optimized to overcome existing issues. However, the sugarcane genome is widely known for different chromosome numbers ranging from 100–130, an abundance of transposon/retro-transposon distribution throughout the genome(s), and highly variable ploidy levels for genes, and repetitive elements (occupying about 50% of the sugarcane genome, as discussed earlier). As a result, the sugarcane monoploid genome could be 10 times larger in size when compared to other model species like *Arabidopsis* (with a genome size of 135 Mb, and *n* = 5 chromosomes) and may contain many paralogous genomic sequences that make sequencing and genome assemblies difficult, which often will generate false-positive errors. Though crops like rice, maize or sorghum, can be of great use in the comparative genomics of sugarcane, the level of polyploidy existing in sugarcane is unmatched. With the available sorghum reference genome, not much is inferred regarding the isoform/allele specific information in sugarcane lacking sufficient annotation which reflects its inadequacy when referenced for sugarcane. Thus, the application of the genomics research approaches in sugarcane is challenging the existing knowledge of polyploidy and its management in the context of genome sequencing.

The ratio of the chromosomal inheritance in a hybrid may not be consistent and every hybridization results in a new genetic composition. In sugarcane, basic cytogenetic information for each species or cultivar is highly uncertain and debated, due to the inherent methodological difficulties in precisely ascertaining the chromosome numbers that are confined within the nucleus. The recent advances in molecular cytogenetics has helped to better understand the origin of sugarcane, as until recently only plant morphology and cytogenetics were employed in the taxonomic classification of sugarcane.

The first step towards a genome sequence for a crop species is the production of a suitable reference assembly. In the current genomics context, a single genome sequence of a plant species does not reflect the complete genetic complement available for that species which has now resulted in a new branch of study of “pan-genomes” and “core genomes” (Montenegro et al., 2017). Interestingly, this is very relevant to the case of sugarcane wherein a genomic sequence of a species or cultivar of sugarcane may not fully represent the vast diversity that exists in the germplasm due to the occurrence of huge variations in the genetic composition. The sugarcane genome is highly prone to chromosome eliminations, which might lead to an incomplete genome sequence in a reference/representative sequence for sugarcane hybrids. The presence or absence of genes or genomic regions between individuals of the same species, is an important form of variation in plants, and the sum of core and variable regions of the genome for a species (pan-genome) facilitated the wheat, rice, *Brachypodium* and *Brassica* genomic sequencing enormously (Montenegro et al., 2017). With its high ploidy levels and distinct genetic compositions (as there could be genotype specific alleles), creating a reference database or a pan-genome or working with a reference database would be a real challenge in sugarcane. The assembled gene sequences may fail to represent the true sequences, and some of the identical gene families may result in a mosaic of sequences without actually representing any member of the family (Claros et al., 2012) and this is expected to occur frequently in a polyploid genome like sugarcane.

Commercial sugarcane plants are interspecific poly-aneuploid hybrids. Most of the traits in sugarcane are found to be polygenic and are quantitatively inherited (Casu et al., 2005) which still makes the use of molecular markers challenging and to-date, no reliable, reproducible trait specific marker had been developed for sugarcane. The application of molecular markers has had very little impact in sugarcane breeding programs until now. The currently available statistical models have been mostly developed for diploid organisms while the polymorphic loci obtained in sugarcane most often cannot be properly interpreted due to the difficulties of polyploid segregation (Garcia et al., 2006, 2013). Thus, an improvement in the application of statistical models to best fit the complex genetic system of sugarcane is much needed. Further, the available software and next generation sequencing technologies are mainly based on diploids and the variant calling feature in many of the genome variant detection programs does not give reliable results (Sandmann et al., 2017). This is especially complex for sugarcane which requires a genome coverage as high as possible by the existing technology to resolve allelic variation existing in the sugarcane genome (Margarido and Heckerman, 2015). In addition to this, sugarcane having 8–14 alleles confounds the fact that the allelic variation can easily be considered as sequencing error and error corrections using one single isoform/allele as a reference would certainly result in the loss of precious polymorphism existing between two samples.

Despite recent advances in biotechnology for other related crops like sorghum, maize, rice, etc., sugarcane remains an enigma in the genomics context. The sugarcane genome is not sequenced yet nor there are well-annotated transcriptome datasets available, though efforts are underway toward it (SUGESI, 2017). Though wheat has a larger genome than sugarcane, the availability of clear demarcations of the sub-genome (A, B, D, which are diploid progenitors) specific chromosomes made the sequencing easier whereas, sugarcane has polyploid progenitors to begin with. The draft genome sequences of bread wheat, and its progenitors *Triticum urartu* (Ling et al., 2013) and *Aegilops tauschii* (Jia et al., 2013) were simultaneously published which made the sequencing of bread wheat and comparative genome analyses much easier. Similarly, in the case of the allo-octoploid cultivated strawberry, *Fragaria vesca* which was a diploid was sequenced to address the problems of polyploidy (Claros et al., 2012). Large genomes including the tetraploid soybean genome which is 1.1 Gb in size (Schmutz et al., 2009), the sorghum genome of 730 Mb similar to that of the monoploid genome of sugarcane (Paterson et al., 2009), the maize genome of 2.3 Gb in size (Schnable et al., 2009) have been sequenced. More recently, the largest ever plant genome sequence (megagenome) at a size of 31 Gb of sugar pine (*Pinus lambertiana*) (Stevens et al., 2016) and the genome of the wild emmer *Triticum turgidum* at a size of 10.1 Gb (an allotetraploid progenitor of wheat) (Avni et al., 2017) were also published. Thus, currently, the large genome size of sugarcane does not pose a greater challenge but the high ploidy level and the heterozygosity does.

In general, plant genomes are characterized by the presence of large scale duplications and surprisingly it is found to have occurred in even a simple genome like *Arabidopsis* (The Arabidopsis Genome Initiative, 2000). In addition, plant genomes are found to have large proportions of highly repetitive DNA and segmental duplications or WGDs (Levasseur and Pontarotti, 2011) due to polyploidization events, which causes problems in their genome assembly. A high level of duplication of genes or chromosomal segments results in the higher chance of mix up among large genomic fragments (Lin et al., 2000). In sugarcane, this is especially problematic because of the many different homologous and homo(eo)logous chromosomes. This may result in the whole genome sequencing achieved at the expense of “assembly fidelity in repetitive regions and expanded need for computational resources” (Deschamps and Llaca, 2016). Most plant genomes sequenced by NGS are reported to produce “drafts” that are suitable for obtaining gene catalogs, estimating the repetitive content of the genome, establishing the phylogeny and evolutionary relationships, and performing comparative genomics (Claros et al., 2012).

### The Sugarcane Genome Sequencing Strategies and Progress

The availability of a well annotated reference genome would provide fundamental tools for high-throughput re-sequencing and opportunities for extending our knowledge of the plants domestication history and thereby accelerating crop improvement (Morrell et al., 2012; Jiao et al., 2017). Many reference genomes of important crops have been constructed in recent years, most of which were based upon short-read sequencing platforms and often fragmented with the complex repeat regions computationally collapsed (Jiao et al., 2017). The sugarcane genome is far from being complete due the extremely complex nature of the genome. As discussed earlier, the challenges in sequencing of the sugarcane genome lies in the high repetitive content, high levels of polyploidy and heterozygosity, in which the genome contains homo(eo)logous chromosomes originating from two different progenitors. The short-read sequencing and assembly cannot resolve the issues and normally generates incomplete and unplaced contigs which can be as much as hundreds of thousands in number. It is also believed that short-read based assembly reduces the complexity of the genome by collapsing the highly similar sequences and repetitive content into single contigs (Green, 2002; Treangen and Salzberg, 2011). The sugarcane genome could have about the same or even more repetitive content than the sorghum genome (Jannoo et al., 2007; Souza et al., 2011). Ignoring the repetitive content and focussing on the gene-rich regions of the genome could result in missing important biological phenomena which could be crucial for dissecting the functional aspects of the sugarcane genome (Treangen and Salzberg, 2011). The advances in genome sequencing and the emergence of the long-read technology potentially would aid in the completion of the sugarcane genome sequencing.

**FIGURE 2:**
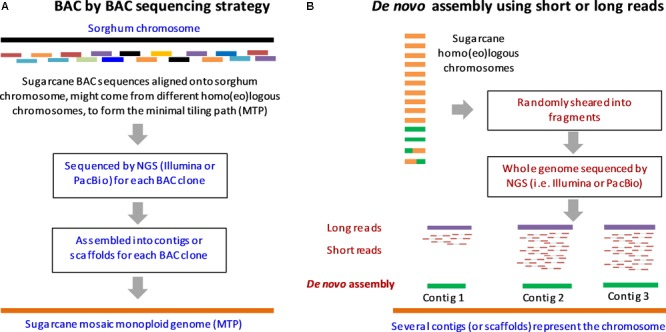
Sequencing strategies proposed for sugarcane genome. **(A)** BAC by BAC sequencing strategy. **(B)** Whole genome shotgun sequencing by short-read and long-read technologies and *de novo* assembly. To simplify, the assembly of one chromosome (out of 10) is shown here. Adapted from (Aitken et al., 2016; Garsmeur et al., 2017).

The Sugarcane Genome Sequencing initiative (SUGESI) has selected the French cultivar R570 (2n = 115) for sequencing as it is the most “intensively characterized” cultivar in recent times (Souza et al., 2011; Aitken et al., 2016). The genomic resources available for this cultivar include genomic sequences (i.e., Kim et al., 2013; Berkman et al., 2014), a high density map (Rossi et al., 2003) and a BAC library (Tomkins et al., 1999). These should facilitate sequencing and assembly of the first sugarcane reference sequence. Other cultivars (including the Brazilian cultivar SP80-3280 which has a lesser contribution from *S. spontaneum*) and the Australian cultivar Q165 have also been studied intensively; and could potentially be used for whole genome re-sequencing and assembly based upon short-read technologies, once the first draft genome of the selected R570 is made available (Manners, 2011; Souza et al., 2011; Aitken et al., 2016). As mentioned earlier, the use of the less complex genomes of the autopolyploid progenitors could simplify sequencing and help identify the original genomic contribution of each progenitor in the hybrid. The progenitor cultivars including *S. officinarum* cultivar LA Purple, cultivar IJ76-514, and *S. spontaneum* cultivar SES208, cultivar Mandalay were used in genome sequencing survey, genetic mapping and BAC library construction (Manners, 2011; Souza et al., 2011; Berkman et al., 2014). Progress has been made in the sugarcane genome sequencing based upon BAC sequencing, short-read and long-read technologies. **Figure 2** summarizes the sequencing strategies proposed for the sugarcane genome, employing two approaches, a BAC by BAC sequencing and *de novo* assembly using short-read and long-read data.

### BAC by BAC Sequencing Strategy

Currently, the BAC by BAC sequencing strategy is believed to be the most reliable approach for sequencing of highly repetitive, polyploid and homo(eo)logous genomes such as that of sugarcane, to overcome the limitations of the short-read/or long-read technologies being used alone (Eversole et al., 2009; Steuernagel et al., 2009; Feuillet et al., 2011). This strategy was applied successfully to many plant genome sequencing projects including *Arabidopsis* (The Arabidopsis Genome Initiative, 2000), rice (Goff et al., 2002) and maize (Schnable et al., 2009). Even though, BAC by BAC sequencing is reliable for construction of the first reference genome compared to the whole genome shotgun sequencing, it is a costly approach, especially for a large genome composed of homo(eo)logous chromosomes (Paterson et al., 2010; Manners, 2011). BAC sequencing of the sugarcane genome is currently in progress (Aitken et al., 2016).

A BAC library constructed for the hybrid cultivar R570 includes 103,296 clones of an average insert size of 130 kb, estimated to be 14X of the monoploid genome or about 1.3X of the whole (heterozygous) genome (Tomkins et al., 1999; Grivet and Arruda, 2002). About 5,000 BAC clones were selected from the R570 BAC library based on those clones anchored to the sorghum genome, to help the assembly of a monoploid genome coverage (minimal tiling path) of the sugarcane genome in the on-going SUGESI (Souza et al., 2011; SUGESI, 2017). It is important to note that due to the high heterozygosity of the sugarcane genome, this minimal tiling path includes BAC clones that might have come from different chromosomes in the homo(eo)logous groups aligned onto the sorghum genome, which forms a mosaic monoploid genome of sugarcane (Paterson et al., 2010; Garsmeur et al., 2017). This means that the resultant mosaic assembly, which was shown to be feasible to construct thanks to the high micro-collinearity amongst the sugarcane homo(eo)logous chromosomes (Jannoo et al., 2007; Garsmeur et al., 2011), would not reflect the allelic variation within the homo(eo)logs. This was proposed due to the fact that sequencing of all BAC clones efficiently covering all homo(eo)logous chromosomes in the sugarcane (which could be up to a million clones) would require a huge investment and tremendous efforts to achieve (Paterson et al., 2010). However, the analysis of the allelic variation could be performed once the first mosaic genome sequence is available (Manners, 2011).

As of 2017, a total of 2,767 BAC clones were sequenced by different groups within SUGESI including CSIRO, University of Queensland (Australia), University of Sao Paolo (Brazil), The South African Sugarcane Research Institute - SASRI (South Africa) and French Agricultural Research Centre for International Development - CIRAD (France) (Aitken et al., 2016; SUGESI, 2017). It is proposed that the rest of the 5,000 BAC clones will be sequenced to complete the minimal tiling path of the sugarcane genome (Aitken et al., 2016). It is noteworthy to mention that this takes only the BAC clones anchored onto the sorghum genome, which means only BAC clones containing conserved genes collinear between sugarcane and sorghum were used. Most selected BAC sequences were obtained from short-read technologies (i.e., 2 × 250 bp from Illumina Hi-Seq 2500). As a result, the assembled sequences contain a portion of BAC clones represented in more than one contig or scaffold which could be due to the uneven sequencing depth or repetitive content within the BAC sequences. With the advent of the third-generation sequencing technologies, the use of the long-read in improving of BAC clone sequencing and assembly will be discussed in the next section.

### Whole Genome Sequencing by Second Generation Sequencing and *de novo* Assembly

The advance in next generation sequencing technology (second generation), together with a sharp reduction in genome sequencing cost (as reviewed in van Dijk et al., 2014) allow whole genome sequencing generating short-read data for a species, even for complex genomes like sugarcane, at low costs in a short time. The challenges in sugarcane genomics lie in the assembly of the fragmented genome (reads) into complete chromosome sequences which has been impeded due to the high amount of repetitive content, high levels of polyploidy and heterozygosity. To overcome this, quite often, enrichment of coding regions of the sugarcane genomic DNA was used to ensure an efficient coverage depth captured, and minimize the effect of repetitive content on the analysis in the context of a lack of a reference sequence (Bundock et al., 2012; Henry et al., 2012). The limitation in read length of this technology results in the collapse of the reads originally from repetitive sequences and hence reduces the genome complexity and the genome completeness (Green, 2002; Treangen and Salzberg, 2011). Moreover, ambiguous bases are normally introduced into the assembly through scaffolding and these assemblies are mostly represented in unplaced scaffolds which are not represented in chromosomes. The second generation sequencing technologies were mostly applied for purposes such as allelic variation analysis within the “gene-rich regions” of the sugarcane genome by sequencing of enriched DNA fragments (Bundock et al., 2009; Bundock et al., 2012), or sequencing of enriched euchromatic regions of the genome by methylation filtration (Grativol et al., 2014) or low coverage whole genome surveying and allelic diversity study (Berkman et al., 2014). Conglomerate monoploid assemblies for three sugarcane hybrid cultivars (Q165, R570 and SP80-3280) and three progenitor cultivars (IJ76-514, LA Purple and Mandalay) based upon Illumina short-reads (length of 76 bp) were obtained for estimation of genome size, repetitive content (by k-mer count approach) and SNP polymorphism detection in the *Saccharum* genomes (Berkman et al., 2014). The first short-read derived genome assembly constructed by Aitken et al. (2016) contains 830 Gb data in 4,083,536 assembled scaffolds, which equates to 83 times of the total sugarcane genome. This confirms the difficulty of assembling such a complex polyploid genome. The assembly covers about 67% of genome sequence of the sugarcane cultivar R570. The version 0.1 of the assembly (Sugarcane v0.1 GBrowse) can be accessed from CSIRO server http://gbrowse-ext.bioinformatics.csiro.au/gb2/gbrowse/shybrid0.1/, which allows users to perform homology search and view the regions of interest based on alignment of the scaffolds on the sorghum genome.

The exploitation of second generation sequencing technologies in *de novo* construction of the sugarcane genome might not be feasible considering the short-read length compared to the repetitive regions and the lack of a reference sequence. However, it can be used in such a hybrid manner to aid in BAC by BAC sequencing strategy or *de novo* assembly based on long-read technologies (i.e., in error correction). Once the genome sequence is available, thanks to the great depth and low error rate that the second-generation sequencing platforms offers, it would play important roles in re-sequencing of different cultivars/varieties of interest, or polymorphism analysis to evaluate the allelic variation in the sugarcane genome.

### Long-Read From Third Generation Sequencing Technology to Aid Genome Assembly

The high heterozygosity and repetitive content in the sugarcane genome suggest that the read length plays an important role in achieving assembly completeness. Third generation sequencing technologies offer longer reads, faster results and simpler library preparation compared to the second generation (Bleidorn, 2016). However, these techniques require very high quality high molecular weight (HMW) DNA. The currently available third generation long-read sequencing technologies including PacBio Single molecule real time (SMRT) sequencing (Eid et al., 2009), the Illumina Tru-Seq Synthetic Long-Read technology (McCoy et al., 2014) and the Oxford Nanopore Technologies sequencing platform (Branton et al., 2008; Clarke et al., 2009; Loman et al., 2015) produce an average read length between 5 and 20 kb, and can reach up to 100 kb (Lee et al., unpublished). The availability of long-read technology significantly improves *de novo* genome assembly (Koren and Phillippy, 2015), especially, potentially for those genomes with high and long repeat sequences which are normally not possible to resolve by short-read assemblers (using k-mer approach) (Bleidorn, 2016). These emerging technologies switch the *de novo* assembly approach from k-mer based to overlap-layout-consensus, to self-correct and generate the longer consensus sequences without fragmentation of the reads into k-mers prior to assembly (Miller et al., 2010; Bleidorn, 2016). The latest PacBio RS II and SMRT sequencing by Sequel Systems now can generate read length of up to around 60 kb and ∼10 Gb data per SMRT cell, while algorithms have been developed to improve the per-base accuracy caused by a higher error rate of this platform (PacBio, 2016). The Illumina Tru-Seq Synthetic Long-Read technology can produce read lengths of ∼10 kb, and at the same time offers a very high accuracy (∼0.1% error rate), but can suffer chemistry bias (Lee et al., unpublished). The Oxford Nanopore MinION handheld device offers read length as long as those from PacBio platform, however, it has a high error rata and low throughput (Loman et al., 2015; Lee et al., unpublished). In general, these technologies are currently still very costly to apply.

The PacBio RS II has been tested in sequencing of the sugarcane genome (Lee et al., 2015). At the time of writing this review, the first draft genome assembly obtained from the Illumina Tru-Seq Synthetic long-read technology was made publicly available for the sugarcane cultivar SP80-3280 (Riaño-Pachón and Mattiello, 2017). The assembly was based on 1,224,061 reads with length ranging from 1.5 to ∼20 kb, to aid the construction of the complex and highly repetitive genome at an estimated genome coverage of 4–5X. It was constructed by Celera Assembler v. 8.2 (Myers et al., 2000), representing 199,028 contigs 1,169,948,913 bp (∼1 Gb) in length, with a contig N50 (N50 can be described as a weighted median statistic such that 50% of the entire assembly is contained in contigs or scaffolds equal to or larger than this value) of 8,451 bp. A total of 300,000 protein coding genes derived from transcriptome data were identified in the assembly and 90% of the assembly was covered by eukaryotic coding genes (Riaño-Pachón et al., 2016). Gene prediction revealed that the assembly contains 153,078 predicted protein coding genes of which 37% matched the PFAM domain database. The assembly is available with GenBank accession GCA_002018215.1 and also for blast homology search via http://bce.bioetanol.cnpem.br/ctbeblast/.

The long-read technologies have potential to play important roles in the completion of reference genomes or in the improvement of currently available genome sequences for many crop species. These technologies could overcome the challenges confounding the genome assembly from short-read data, to generate a more usable and complete reference sequence. For instance, in a recent study, Jiao et al. (2017) employed the PacBio single-molecule technologies in combination with high-resolution optical mapping to improve the assembly and annotation of maize inbred line B73 reference genome. This resulted in a 52-fold increasing in contig length and improvements in the assembly of repetitive, intergenic and centromeric regions of the new sequences compared to the previous reference genome. Moreover, a hybrid approach of combining the advantages the short and long-read technologies seems to be potentially useful in reducing the sequencing cost per genome to obtain a certain level of coverage solely from the long-read technologies, yet with increased assembly accuracy. For example, using reads from the Illumina platform can aid in error correction of assembled contigs generated from PacBio (Koren et al., 2012) or Oxford Nanopore technologies (Madoui et al., 2015). The use of a PacBio RS II instrument in sequencing selected BAC clones through international collaboration in the SUGESI consortium was also proposed, to obtain 100X depth of coverage (Garsmeur et al., 2017). This aims to sequence a core set of 4,688 sugarcane selected BAC clones representing a mosaic of the basic monoploid genome in high quality, and about 86% of BACs assembled in one contig each and covering ∼80% of the sorghum genome (Garsmeur et al., 2017).

## Comparative Genomics

Comparative genomics is considered as a powerful tool to accelerate progress in studying the “genomic structure of crops that are lacking in the necessary genomic tools” (Aitken et al., 2014). The discovery of very high levels of similarity of gene order (collinearity) among grasses and more distantly related taxa led to the dissection of larger genomes using the available genetic information from relatedly smaller genomes. The sequencing of the rice genome in 2002 (Goff et al., 2002; Yu et al., 2002), maize genome in 2009 (Schnable et al., 2009) followed by a number of other crop genome sequences like sorghum and wheat heralded a new era of functional genomics in the grasses. Novel approaches to perform expression based quantitative trait locus analysis, genome-wide association studies, transposons and repetitive content estimation, mining rare allelic variants and identifying insertional mutants underlying agronomically important traits were developed. Comparative genomic studies among rice, maize, and sorghum, was made possible by the availability of sequenced genomes and their close evolutionary history among these species. The relatively small sorghum genome has become an important reference source for closely related large-genome crops such as maize, sugarcane and the distantly related rice genome, however, sorghum is more closely related to maize, than rice to maize and sugarcane (Draye et al., 2001). Further, it was reported that as the diploid progenitors for sugarcane is not known, sorghum can be easily considered in its place (Al-Janabi et al., 1994). A near perfect marker collinearity was observed between sorghum and sugarcane and it was predicted that orthologous allele could be cloned from sugarcane using sorghum genome map positions (Guimarães et al., 1997). Maize and sorghum are reported to have had a common ancestor as recently as about 11.9 MYA, after which, maize underwent an ancient tetraploidization (Swigoňová et al., 2004). The maize genome is said to contain 3.4 times the DNA content of sorghum and 6.3 times the DNA content of rice, and it has a very well annotated genome compared to sorghum^2^.

In addition to sorghum, maize genomic information is a valuable resource for comparative studies in sugarcane due to the detailed annotations available for this genome. Rice is another grass sharing a common ancestor with maize-sorghum about 50–80 MYA and is distantly related to sugarcane. The sequences of grasses like *Setaria* and *Brachypodium* may also facilitate comparative genomics of sugarcane to some extent. *Brachypodium* belongs to the Pooideae sub-family and a draft genome sequence was completed and released in 2008. *Setaria* is a millet crop and is much more closely related to many of the bioenergy grasses, including maize, sorghum, *Miscanthus* spp., switchgrass, and sugarcane than to *Brachypodium* (Brutnell et al., 2015). The other sugar accumulating crop, sugar beet (*Beta vulgaris*) was sequenced (Dohm et al., 2014), however, little does it help sugarcane comparative studies as it is a dicot with a genome size of 714–758 Mb. There are various databases available for comparative genomics of sugarcane with related crops. The South Green bioinformatics platform^3^, phytozome^4^, Ensembl Plants datasets in direct collaboration with *Gramene*^5^ are some of the few to be mentioned.

### Evolutionary History of Sugarcane

**FIGURE 3:**
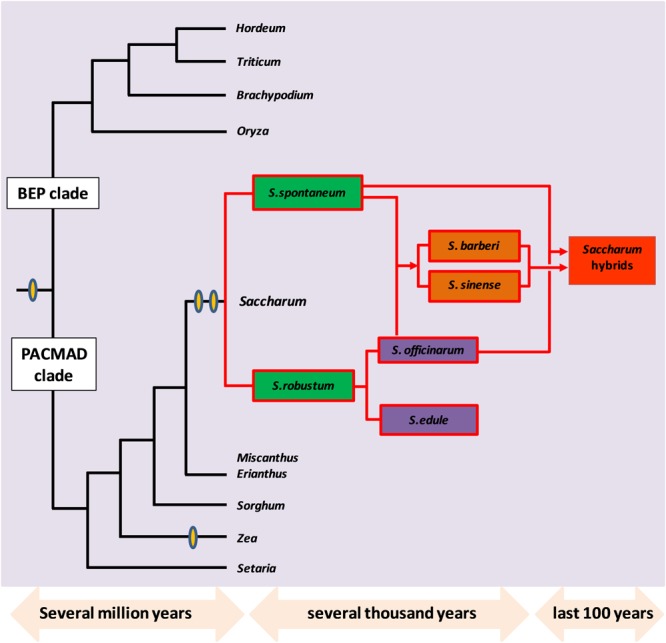
Evolutionary history of sugarcane (*Saccharum* hybrids) in comparison with progenitor species *S. officinarum, S. spontaneum, S. robustum, S. sinense, S. edule* and other related genera in PACMAD and BEP clades. The whole genome doubling events are denoted by yellow ovals. Figure adapted and redrawn from (D’Hont et al., 2008; Metcalfe et al., 2015).

Sugarcane species belong, to the sub-tribe Saccharinae, in the tribe Andropogoneae which includes the other C4 crops like sorghum, *Miscanthus* and *Zea mays* (Paterson et al., 2013). Modern sugarcane hybrids are derived from different interspecific crosses of the *Saccharum* complex that includes *S. officinarum, S. spontaneum, S. sinense, S. edule*, and *S. barberi* and also through genetic contribution from other related genera like *Miscanthus, Erianthus*, and *Sclerostachya* (Paterson, 2012). *S. spontaneum* is reported to be originated in India while *S. officinarum* originated from Papua New Guinea (Grassl, 1977; Roach and Daniels, 1987; Amalraj and Balasundaram, 2006). Many members of the *Saccharum* complex frequently interbreed producing intermediate forms, that are euploids and aneuploids with some of them having a new genome structure due to different types of chromosome transmission (Paterson et al., 2012). Traditionally cytological and morphological characters were used for defining probable evolutionary relationships within Saccharinae. With the recent advances in molecular markers and genomics-based techniques, the evolutionary aspects of the *Saccharum* complex and its related genera are beginning to be explored without any ambiguities. Chloroplast DNA, mitochondrial and nuclear ribosomal gene markers were applied to establish the probable polyphyletic origins of *Saccharum* with sorghum, *Erianthus, Miscanthus*, and other related genera (D’Hont et al., 1993, 1995; Selvi et al., 2005; Tambarussi et al., 2009; Singh et al., 2010; Viola et al., 2011; Zhu et al., 2014; Raj et al., 2016). It was found that the closest sugarcane diploid relative that could be identified till date, is *Narenga porphyrocoma* (Al-Janabi et al., 1993), which had diverged from sugarcane at 2.5 MYA, while *S. spontaneum* and *S. officinarum* diverged at 1.5–2 MYA (Garsmeur et al., 2011). *Miscanthus* species have a basic chromosome set of *n* = *x* = 19 (Swaminathan et al., 2012), while many of the *Saccharum* species have *x* = 10 that is characteristic of several Saccharinae species (D’Hont and Glaszmann, 2001). The transition from *x* = 10 to 19 in *Miscanthus* might be due to a polyploidization event that occurred 8–9 MYA since its divergence from sorghum. However, while *Saccharum*, has polysomic transmission of chromosomes, *Miscanthus* is reported to have a preferential pairing of chromosomes (Paterson, 2012). With respect to other related genera like rice, maize and sorghum, it is estimated that sugarcane diverged from sorghum around 6–9 MYA and the divergence of sorghum and rice occurred around 43 MYA (Paterson et al., 2004; Jannoo et al., 2007). Rice and the maize/sorghum lineages could have diverged from a common ancestor about 66 MYA, having higher levels of chromosome structural rearrangement. Sorghum and sugarcane are reported to have shared a common ancestor as recently as 5 MYA, sharing high collinearity and producing viable progeny in intergeneric crosses (Draye et al., 2001; Nair et al., 2006). **Figure 3** summarizes the evolutionary history of sugarcane (*Saccharum* hybrids) in comparison with its progenitor species, and other related genera in PACMAD and BEP clades.

### Studies on Collinearity and Synteny of Sugarcane With Related Crops

Many species in the grass family, especially rice, maize and sorghum, have genomes that were well characterized much earlier than that of sugarcane. Hence, understanding the co-linearity and synteny of sugarcane with these related grass species could benefit sugarcane genomic studies (Grivet and Arruda, 2002). It is evident that there exists a very high level of micro-collinearity among the homo(eo)logous chromosomes in the sugarcane genome, which is due to the two parental species being very closely related (Jannoo et al., 2007; Garsmeur et al., 2011). Amongst grass species, rice is the best characterized and could be a beneficial model species for sugarcane, despite not being the most related species to sugarcane, or the species with the highest collinearity with sugarcane (Paterson et al., 1995; Glaszmann et al., 1997; Grivet and Arruda, 2002). Many rearrangements and a relatively global synteny between the sugarcane and rice genomes were reported in Glaszmann et al. (1997) due to the large distance between the two species. Several sources of genomic information (Goff et al., 2002; Yu et al., 2002) together with gene models (Ouyang et al., 2007; Tanaka et al., 2008) were available for rice earlier than for most of the other grasses, which could facilitate sugarcane genomic studies. Comparative studies between sugarcane and maize revealed that most co-linearity between the two genomes is also rearranged and altered (at most loci, one locus in sugarcane is orthologous to two loci in the maize genome), which could have resulted from segmental allopolyploidy and diploidization events that occurred after the divergence of maize from sugarcane (Grivet et al., 1994; Dufour et al., 1996; Gaut et al., 2000). A maize reference genome (Schnable et al., 2009; Jiao et al., 2017) and gene models (Andorf et al., 2016) are also available. Compared to maize, sorghum has a shorter divergence time from sugarcane, therefore, sugarcane has a good micro-collinearity and simplest synteny with sorghum (Dufour et al., 1996, 1997; Glaszmann et al., 1997; Guimarães et al., 1997; Ming et al., 1998; Wang et al., 2010; Figueira et al., 2012). For this reason, sorghum is believed to be the best diploid and the most closely related species to sugarcane to be used as a reference for sugarcane studies (Grivet et al., 1994; Grivet and Arruda, 2002; Dillon et al., 2007). The sorghum genome sequence (Paterson et al., 2009) and gene models (PlantGDB, 2017) have been intensively used in sugarcane research.

### Sorghum Genome as the Closest Reference

The first genome sequence for sorghum was based on the standard Sanger sequencing methodologies on cultivar BTx623 at a coverage of ∼8.5X (Paterson et al., 2009). This sorghum genome version 1 has 10 *pseudo*-molecules (corresponding to the 10 chromosomes) represented in 6,929 contigs, with 659.2 Mb total scaffolds (625.6 Mb of genomic sequence), an N50 of 64.3 Mb and an estimated error rate of <1 per 10 kb (Paterson et al., 2009; McCormick et al., 2018). About 27,640 protein-coding genes were annotated, out of 34,496 gene models predicted for sorghum, of which 24% were found to be grass-specific while 7% were unique to sorghum (Paterson et al., 2009). Using this genome assembly from cultivar BTx623 as a reference, genomes from several other genotypes have been re-sequenced and assembled based on read mapping approach, for examples, genotypes BTx642 and Tx7000 (Evans et al., 2013, 2017) and 44 sorghum genotypes (Mace et al., 2013).

Recent improvements have been made to the assembly and annotation of the sorghum genome version 1 by deep whole genome shotgun sequencing (110X) (McCormick et al., 2018). The most updated sorghum genome (version 3) has improved genome organization, with an addition of 29.6 Mb of genomic information added to the existing assembly, a total of 34,211 genes annotated, increased average gene length and N50 and less errors. The total number of contigs in this version was reduced to 2,688, while total scaffold sequences was 683.6 Mb (655.2 Mb of genomic sequence), scaffold N50 was 68.7 Mb and estimated error rate was <1 per 100 kb (McCormick et al., 2018). The genome size estimated for sorghum by flow cytometry was about 818 Mb (Price et al., 2005), suggesting that the reference genome version 3 represents about 80% of the total genome. The sorghum genome contains ∼61% repetitive content, a high gene parallelism with the sugarcane genome, and less gene duplication in comparison with other C4 grass species (Paterson et al., 2009, 2010; Paterson, 2012).

## Future Prospects

Sequencing plant genomes has become a laboratory routine and currently reference genomes are available for non-model and under explored plants. Many of these genomes have been long neglected due to the high costs and facilities that were required previously. The sugarcane genome has received a greater interest in recent times owing to its economic value in the context of second generation bioenergy production (Cheavegatti-Gianotto et al., 2011). For about the past 100 years, a few interspecific crosses between *S. officinarum* and *S. spontaneum* have resulted in the development of sugarcane hybrid genotypes with a very narrow genetic base. There is wide spread recognition for the need to widen the germplasm incorporated in the conventional hybrids to meet the current demands in today’s agriculture. The basic knowledge of the sugarcane genome and its complexities had been made available through cytological studies, and molecular markers. A number of recent studies, using high throughput techniques from Sanger to next-generation sequencing of sugarcane cultivars and wild *Saccharum* species though have not attained completion, have improved our understanding of gene and repetitive contents of the genome and allelic variation. About 3.7% of the monoploid genome was sequenced using BAC-based approach which helped in establishing a framework for the sugarcane genome annotation and phylogenetic studies and the methylation pattern of the sugarcane was studied through a methylation filtration sequencing approach (de Setta et al., 2014; Grativol et al., 2014; Metcalfe et al., 2015) apart from the development of large scale EST collection for functional studies (Vettore et al., 2001), the genetic map construction (Rossi et al., 2003; Aitken et al., 2005, 2014; Garcia et al., 2006) and BAC libraries (Tomkins et al., 1999).

Despite the advances in sequencing technology, and several sequencing initiatives in the last few years such as the BAC by BAC approach and the ongoing whole genome shotgun sequencing of sugarcane, the outcome is slower compared to that with other crops, due to the large size and complexity of the sugarcane genome. The availability of sorghum and maize genomes to certain extent has allowed an increase in genomic studies in sugarcane. Currently the publicly available resources for sugarcane are the *Saccharum officinarum* gene indices version 3, which includes ESTs and other assembled transcriptome sequences (SoGI, 2017); other published transcriptome data, i.e., Cardoso-Silva et al. (2014) and (Hoang et al., 2017) and the first draft genome sequences of the sugarcane hybrid SP80-3280 (Riaño-Pachón and Mattiello, 2017). A summary of currently available genomic resources for sugarcane research as of July 2017 is presented in **Table 1**. With all the progress that has been made so far, the assembling of the sugarcane genome might still be challenging due to its inherent genetic complexity. The available sugarcane genome assemblies are highly fragmented, with a low genome completeness, mainly focused on gene-rich regions, while there is little information available for the complex repeats. Advancements in the currently available techniques and new methodologies, like mate-pair libraries, complementing physical assemblies with genetic maps, optical mapping (Deschamps and Llaca, 2016) hopefully would help overcome these issues with sugarcane and a true comparative and structural analyses among its species and cultivars is possible in the near future.

**Table 1:** A summary of currently available resources for sugarcane genomics as of 2017.

Data types	Data description and reference
Genomic sequences	- Draft genome of cultivar SP80-3280 (Riaño-Pachón et al., 2016; Riaño-Pachón and Mattiello, 2017) - Genome assembly of cultivar R570 (Aitken et al., 2016) - Surveyed genome sequences of several cultivars (Berkman et al., 2014)
Organellar genomes	- Chloroplast genome, cultivar NCo310 (Asano et al., 2004) - Chloroplast genome, cultivar SP80-3280 (Calsa Junior et al., 2004) - Chloroplast genome, cultivar Q155 (Hoang et al., 2015b) - Chloroplast genome, cultivar RB867515 (Vidigal et al., 2016) - Mitochondrial genomes cultivar Khon Kaen 3 (Shearman et al., 2016)
ESTs and gene index	- SUCEST database (Vettore et al., 2001; SUCEST-FUN Database, 2015) - *Saccharum officinarum* Gene Index database version 3 (SoGI, 2017)
Transcriptome sequences	- Short-read derived transcriptomes (Cardoso-Silva et al., 2014), (Vicentini et al., 2015), (Li et al., 2016) and (Belesini et al., 2017) - Long-read derived transcriptome (Hoang et al., 2017)
Genetic maps	- High density maps (Rossi et al., 2003), (Aitken et al., 2005), (Garcia et al., 2006) - DArT (Aitken et al., 2014)
BAC libraries	- Cultivar R570 (Tomkins et al., 1999) - Cultivar SP80-3280 (Figueira et al., 2012; Okura et al., 2012) and other cultivars (Souza et al., 2011)
Genomes, transcripts and gene models from close related species	- Sorghum genome version 3 (Paterson et al., 2009; McCormick et al., 2018) - Sorghum transcripts and genome models (PlantGDB, 2017)

## Author Contributions

PT and NH prepared the first draft. RH, NH, and PT critically revised the manuscript. All authors read and approved the final manuscript.

## Conflict of Interest Statement

The authors declare that the research was conducted in the absence of any commercial or financial relationships that could be construed as a potential conflict of interest. The reviewer GB declared a shared affiliation, with no collaboration, with one of the authors RH to the handling Editor.

## Footnotes

1 http://www.divseek.org/

2 https://www.maizegdb.org/

3 http://southgreen.cirad.fr/

4 https://phytozome.jgi.doe.gov/pz/portal.html

5 http://plants.ensembl.org/index.html

## References

[B1] Abberton M. Batley J. Bentley A. Bryant J. Cai H. Cockram J. (2016). Global agricultural intensification during climate change: a role for genomics. 14 1095–1098. 10.1111/pbi.12467 26360509 PMC5049667

[B2] Abdurakhmonov I. Y. (ed.) (2016). “Genomics era for plants and crop species – advances made and needed tasks ahead,” in (Rijeka: InTech). 10.5772/62083

[B3] Aitken K. Berkman P. Rae A. (2016). The first sugarcane genome assembly: How can we use it? 38 193–199.

[B4] Aitken K. Jackson P. Mcintyre C. (2005). A combination of AFLP and SSR markers provides extensive map coverage and identification of homo(eo)logous linkage groups in a sugarcane cultivar. 110 789–801. 10.1007/s00122-004-1813-7 15700149

[B5] Aitken K. Jackson P. Piperidis G. Mcintyre L. (2004). “QTL identified for yield components in a cross between a sugarcane (*Saccharum* spp.) cultivar Q165A and a *S. officinarum* clone IJ76-514”, in Brisbane, QLD.

[B6] Aitken K. S. Mcneil M. D. Hermann S. Bundock P. C. Kilian A. Heller-Uszynska K. (2014). A comprehensive genetic map of sugarcane that provides enhanced map coverage and integrates high-throughput Diversity Array Technology (DArT) markers. 15:152. 10.1186/1471-2164-15-152 24564784 PMC4007999

[B7] Al-Janabi S. M. Honeycutt R. J. Mcclelland M. Sobral B. W. S. (1993). A genetic linkage map of *Saccharum spontaneum* L. ‘SES208’. 134 1249–1260. 8375659 10.1093/genetics/134.4.1249 PMC1205592

[B8] Al-Janabi S. M. Mcclelland M. Petersen C. Sobral B. W. S. (1994). Phylogenetic analysis of organellar DNA sequences in the Andropogoneae: Saccharinae. 88 933–944. 10.1007/BF00220799 24186245

[B9] Amalraj V. A. Balasundaram N. (2006). On the taxonomy of the members of ‘*Saccharum* complex’. 53 35–41. 10.1007/s10722-004-0581-1

[B10] Andorf C. M. Cannon E. K. Portwood J. L. II Gardiner J. M. Harper L. C. Schaeffer M. L. (2016). MaizeGDB update: new tools, data and interface for the maize model organism database. 44 D1195–D1201. 10.1093/nar/gkv1007 26432828 PMC4702771

[B11] Asano T. Tsudzuki T. Takahashi S. Shimada H. Kadowaki K. (2004). Complete nucleotide sequence of the sugarcane (*Saccharum officinarum*) chloroplast genome: a comparative analysis of four monocot chloroplast genomes. 11 93–99. 10.1093/dnares/11.2.93 15449542

[B12] Avni R. Nave M. Barad O. Baruch K. Twardziok S. O. Gundlach H. (2017). Wild emmer genome architecture and diversity elucidate wheat evolution and domestication. 357 93–97. 10.1126/science.aan0032 28684525

[B13] Babu C. Koodalingam K. Natarajan U. Shanthi R. Govindaraj P. (2010). Genetic enhancement of sugarcane (*Saccharum* sp. hybrids) for resistance to red rot disease and economic traits. 4 97–107. 10.4038/jas.v4i3.1648

[B14] Belesini A. A. Carvalho F. M. S. Telles B. R. De Castro G. M. Giachetto P. F. Vantini J. S. (2017). De novo transcriptome assembly of sugarcane leaves submitted to prolonged water-deficit stress. 16 1–20. 10.4238/gmr16028845 28549198

[B15] Berkman P. J. Bundock P. C. Casu R. E. Henry R. J. Rae A. L. Aitken K. S. (2014). A survey sequence comparison of *Saccharum* genotypes reveals allelic diversity differences. 7 71–83. 10.1007/s12042-014-9139-3

[B16] Bleidorn C. (2016). Third generation sequencing: technology and its potential impact on evolutionary biodiversity research. 14 1–8. 10.1080/14772000.2015.1099575

[B17] Branton D. Deamer D. W. Marziali A. Bayley H. Benner S. A. Butler T. (2008). The potential and challenges of nanopore sequencing. 26 1146–1153. 10.1038/nbt.1495 18846088 PMC2683588

[B18] Brutnell T. P. Bennetzen J. L. Vogel J. P. (2015). Brachypodium distachyon and *Setaria viridis*: model genetic systems for the grasses. 66 465–485. 10.1146/annurev-arplant-042811-105528 25621515

[B19] Bundock P. C. Casu R. E. Henry R. J. (2012). Enrichment of genomic DNA for polymorphism detection in a non-model highly polyploid crop plant. 10 657–667. 10.1111/j.1467-7652.2012.00707.x 22624722

[B20] Bundock P. C. Eliott F. G. Ablett G. Benson A. D. Casu R. E. Aitken K. S. (2009). Targeted single nucleotide polymorphism (SNP) discovery in a highly polyploid plant species using 454 sequencing. 7 347–354. 10.1111/j.1467-7652.2009.00401.x 19386042

[B21] Calsa Junior T. Carraro D. M. Benatti M. R. Barbosa A. C. Kitajima J. P. Carrer H. (2004). Structural features and transcript-editing analysis of sugarcane (*Saccharum officinarum* L.) chloroplast genome. 46 366–373. 10.1007/s00294-004-0542-4 15526204

[B22] Cardoso-Silva C. B. Costa E. A. Mancini M. C. Balsalobre T. W. A. Canesin L. E. C. Pinto L. R. (2014). *De novo* assembly and transcriptome analysis of contrasting sugarcane varieties. 9:e88462. 10.1371/journal.pone.0088462 24523899 PMC3921171

[B23] Casu R. E. Manners J. M. Bonnett G. D. Jackson P. A. Mcintyre C. L. Dunne R. (2005). Genomics approaches for the identification of genes determining important traits in sugarcane. 92 137–147. 10.1016/j.fcr.2005.01.029

[B24] Cheavegatti-Gianotto A. De Abreu H. M. C. Arruda P. Bespalhok Filho J. C. Burnquist W. L. Creste S. (2011). Sugarcane (*Saccharum X officinarum*): a reference study for the regulation of genetically modified cultivars in Brazil. 4 62–89. 10.1007/s12042-011-9068-3 21614128 PMC3075403

[B25] Clarke J. Wu H.-C. Jayasinghe L. Patel A. Reid S. Bayley H. (2009). Continuous base identification for single-molecule nanopore DNA sequencing. 4 265–270. 10.1038/nnano.2009.12 19350039

[B26] Claros M. G. Bautista R. Guerrero-Fernández D. Benzerki H. Seoane P. Fernández-Pozo N. (2012). Why assembling plant genome sequences is so challenging. 1 439–459. 10.3390/biology1020439 24832233 PMC4009782

[B27] Cuadrado A. Acevedo R. Moreno Diaz De La Espina S. Jouve N. De La Torre C. (2004). Genome remodelling in three modern *S. officinarumxS. spontaneum* sugarcane cultivars. 55 847–854. 10.1093/jxb/erh093 14990623

[B28] da Silva J. Honeycutt R. J. Burnquist W. Al-Janabi S. M. Sorrells M. E. Tanksley S. D. (1995). *Saccharum spontaneum* L. ‘SES 208’ genetic linkage map combining RFLP- and PCR-based markers. 1 165–179. 10.1007/BF01249701

[B29] Daniels J. Roach B. (1987). “Taxonomy and evolution,” in ed. Heinz D. (Amsterdam: Elsevier Press).

[B30] de Setta N. Monteiro-Vitorello C. B. Metcalfe C. J. Cruz G. M. Del Bem L. E. Vicentini R. (2014). Building the sugarcane genome for biotechnology and identifying evolutionary trends. 15:540. 10.1186/1471-2164-15-540 24984568 PMC4122759

[B31] de Souza A. P. Grandis A. Leite D. C. C. Buckeridge M. S. (2014). Sugarcane as a bioenergy source: history, performance, and perspectives for second-generation bioethanol. 7 24–35. 10.1007/s12155-013-9366-8

[B32] DeBernardi J. E. (2009). Singapore: NUS Press.

[B33] Deschamps S. Llaca V. (2016). “Strategies for sequence assembly of plant genomes,” in ed. Abdurakhmonov I. Y. (Rijeka: InTech). 10.5772/61927

[B34] D’Hont A. (2005). Unraveling the genome structure of polyploids using FISH and GISH; examples of sugarcane and banana. 109 27–33. 10.1159/000082378 15753555

[B35] D’Hont A. Glaszmann J. C. (2001). Sugarcane genome analysis with molecular markers, a first decade of research. 24 556–559.

[B36] D’Hont A. Grivet L. Feldmann P. Rao S. Berding N. Glaszmann J. C. (1996). Characterisation of the double genome structure of modern sugarcane cultivars (*Saccharum* spp.) by molecular cytogenetics. 250 405–413. 10.1007/BF02174028 8602157

[B37] D’Hont A. Ison D. Alix K. Roux C. Glaszmann J. C. (1998). Determination of basic chromosome numbers in the genus *Saccharum* by physical mapping of ribosomal RNA genes. 41 221–225. 10.1139/g98-023

[B38] D’Hont A. Lu Y. Feldmann P. Glaszmann J.-C. (1993). Cytoplasmic diversity in sugar cane revealed by heterologous probes. 1 12–15.

[B39] D’Hont A. Rao P. S. Feldmann P. Grivet L. Islam-Faridi N. Taylor P. (1995). Identification and characterisation of sugarcane intergeneric hybrids, *Saccharum officinarum* x *Erianthus arundinaceus*, with molecular markers and DNA in situ hybridisation. 91 320–326. 10.1007/BF00220894 24169780

[B40] D’Hont A. Souza G.M. Menossi M. Vincentz M. Van-Sluys M.-A. Glaszmann J.C. (2008). “Sugarcane: a major source of sweetness, alcohol, and bio-energy,” in eds Moore P. H. Ming R. (New York, NY: Springer) 483–513. 10.1007/978-0-387-71219-2_21

[B41] Dillon S. L. Shapter F. M. Henry R. J. Cordeiro G. Izquierdo L. Lee L. S. (2007). Domestication to crop improvement: genetic resources for *Sorghum* and *Saccharum* (Andropogoneae). 100 975–989. 10.1093/aob/mcm192 17766842 PMC2759214

[B42] Dohm J. C. Minoche A. E. Holtgrawe D. Capella-Gutierrez S. Zakrzewski F. Tafer H. (2014). The genome of the recently domesticated crop plant sugar beet (*Beta vulgaris*). 505 546–549. 10.1038/nature12817 24352233

[B43] Draye X. Lin Y.-R. Qian X.-Y. Bowers J. E. Burow G. B. Morrell P. L. (2001). Toward integration of comparative genetic, physical, diversity, and cytomolecular maps for grasses and grains, using the *Sorghum* genome as a foundation. 125 1325–1341. 10.1104/pp.125.3.1325 11244113 PMC65612

[B44] Dufour P. Deu M. Grivet L. Dhont A. Paulet F. Bouet A. (1997). Construction of a composite sorghum genome map and comparison with sugarcane, a related complex polyploid. 94 409–418. 10.1007/s001220050430

[B45] Dufour P. Grivet L. D’hont A. Deu M. Trouche G. Glaszmann J. C. (1996). Comparative genetic mapping between duplicated segments on maize chromosomes 3 and 8 and homoeologous regions in sorghum and sugarcane. 92 1024–1030. 10.1007/BF00224044 24166631

[B46] Eid J. Fehr A. Gray J. Luong K. Lyle J. Otto G. (2009). Real-time DNA sequencing from single polymerase molecules. 323 133–138. 10.1126/science.1162986 19023044

[B47] Evans C. Hardin J. Stoebel D. M. (2017). Selecting between-sample RNA-Seq normalization methods from the perspective of their assumptions. 10.1093/bib/bbx008 [Epub ahead of print]. 28334202 PMC6171491

[B48] Evans D. L. Joshi S. V. (2016). Complete chloroplast genomes of *Saccharum spontaneum, Saccharum officinarum* and *Miscanthus floridulus* (Panicoideae: Andropogoneae) reveal the plastid view on sugarcane origins. 14 548–571. 10.1080/14772000.2016.1197336

[B49] Evans J. Mccormick R. F. Morishige D. Olson S. N. Weers B. Hilley J. (2013). Extensive variation in the density and distribution of DNA polymorphism in *Sorghum* genomes. 8:e79192. 10.1371/journal.pone.0079192 24265758 PMC3827139

[B50] Eversole K. Graner A. Stein N. (2009). “Wheat and barley genome sequencing,” in eds Muehlbauer G. J. Feuillet C. (Berlin: Springer) 713–742. 10.1007/978-0-387-77489-3_24

[B51] FAO (2017). Available at: http://www.fao.org/faostat/en/#home [accessed June 25 2017].

[B52] Feuillet C. Leach J. E. Rogers J. Schnable P. S. Eversole K. (2011). Crop genome sequencing: lessons and rationales. 16 77–88. 10.1016/j.tplants.2010.10.005 21081278

[B53] Figueira T. R. Okura V. Rodrigues Da Silva F. Jose Da Silva M. Kudrna D. Ammiraju J. S. (2012). A BAC library of the SP80-3280 sugarcane variety (*Saccharum* sp.) and its inferred microsynteny with the Sorghum genome. 5:185. 10.1186/1756-0500-5-185 22524198 PMC3419638

[B54] Furtado A. Lupoi J. S. Hoang N. V. Healey A. Singh S. Simmons B. A. (2014). Modifying plants for biofuel and biomaterial production. 12 1246–1258. 10.1111/pbi.12300 25431201

[B55] Garcia A. A. Kido E. A. Meza A. N. Souza H. M. Pinto L. R. Pastina M. M. (2006). Development of an integrated genetic map of a sugarcane (*Saccharum* spp.) commercial cross, based on a maximum-likelihood approach for estimation of linkage and linkage phases. 112 298–314. 10.1007/s00122-005-0129-6 16307229

[B56] Garcia A. A. F. Mollinari M. Marconi T. G. Serang O. R. Silva R. R. Vieira M. L. C. (2013). SNP genotyping allows an in-depth characterisation of the genome of sugarcane and other complex autopolyploids. 3:3399. 10.1038/srep03399 24292365 PMC3844970

[B57] Garsmeur O. Aitken K. S. Potier B. Grimwood J. Charron C. Droc G. (2017). “A reference sequence of the monoploid genome of sugarcane [W889],” in San Diego, CA. 10.1038/s41467-018-05051-5 PMC6035169 29980662

[B58] Garsmeur O. Charron C. Bocs S. Jouffe V. Samain S. Couloux A. (2011). High homologous gene conservation despite extreme autopolyploid redundancy in sugarcane. 189 629–642. 10.1111/j.1469-8137.2010.03497.x 21039564

[B59] Gaut B. S. Le Thierry d’Ennequin M. Peek A. S. Sawkins M. C. (2000). Maize as a model for the evolution of plant nuclear genomes. 97 7008–7015. 10.1073/pnas.97.13.7008 PMC34377 10860964

[B60] Glaszmann J. C. Dufour P. Grivet L. D’hont A. Deu M. Paulet F. (1997). Comparative genome analysis between several tropical grasses. 96 13–21. 10.1023/A:1002987620250

[B61] Goff S. A. Ricke D. Lan T. H. Presting G. Wang R. Dunn M. (2002). A draft sequence of the rice genome (*Oryza sativa* L. ssp. *japonica*). 296 92–100. 10.1126/science.1068275 11935018

[B62] Goldstein D. Mintz S. (2015). Oxford: Oxford University Press 10.1093/acref/9780199313396.001.0001

[B63] Gopal L. (1964). Sugar-making in ancient India. 7 57–72. 10.1163/156852064X00030

[B64] Grandont L. Jenczewski E. Lloyd A. (2013). Meiosis and its deviations in polyploid plants. 140 171–184. 10.1159/000351730 23817089

[B65] Grassl C. (1977). The origin of the sugar producing cultivars of *Saccharum*. 39 8–33. 26497505

[B66] Grativol C. Regulski M. Bertalan M. Mccombie W. R. Da Silva F. R. Zerlotini Neto A. (2014). Sugarcane genome sequencing by methylation filtration provides tools for genomic research in the genus *Saccharum*. 79 162–172. 10.1111/tpj.12539 24773339 PMC4458261

[B67] Green P. (2002). Whole-genome disassembly. 99 4143–4144. 10.1073/pnas.082095999 11904394 PMC123614

[B68] Grivet L. Arruda P. (2002). Sugarcane genomics: depicting the complex genome of an important tropical crop. 5 122–127. 10.1016/S1369-5266(02)00234-0 11856607

[B69] Grivet L. D’hont A. Dufour P. Hamon P. Roques D. Glaszmann J. C. (1994). Comparative genome mapping of sugar cane with other species within the Andropogoneae tribe. 73 500–508. 10.1038/hdy.1994.148

[B70] Grivet L. Dhont A. Roques D. Feldmann P. Lanaud C. Glaszmann J. C. (1996). RFLP mapping in cultivated sugarcane (*Saccharum* spp): genome organization in a highly polyploid and aneuploid interspecific hybrid. 142 987–1000. 8849904 10.1093/genetics/142.3.987 PMC1207035

[B71] Guimarães C. T. Sills G. R. Sobral B. W. S. (1997). Comparative mapping of Andropogoneae: *Saccharum* L. (sugarcane) and its relation to sorghum and maize. 94 14261–14266. 10.1073/pnas.94.26.14261 9405600 PMC24932

[B72] Ha S. Moore P. H. Heinz D. Kato S. Ohmido N. Fukui K. (1999). Quantitative chromosome map of the polyploid *Saccharum spontaneum* by multicolor fluorescence in situ hybridization and imaging methods. 39 1165–1173. 10.1023/A:1006133804170 10380803

[B73] Hatch M. Slack C. Johnson H. S. (1967). Further studies on a new pathway of photosynthetic carbon dioxide fixation in sugar-cane and its occurrence in other plant species. 102 417–422. 10.1042/bj1020417 6029601 PMC1270262

[B74] Hatch M. D. (2005). “C4 photosynthesis: discovery and resolution,” in eds Govindjee J. T. Beatty H. Gest Allen J. F. (Dordrecht: Springer) 875–880. 10.1007/1-4020-3324-9_78

[B75] Heinz D. J. (1987). Amsterdam: Elsevier.

[B76] Henry R. J. Edwards M. Waters D. L. Gopala Krishnan S. Bundock P. Sexton T. R. (2012). Application of large-scale sequencing to marker discovery in plants. 37 829–841. 10.1007/s12038-012-9253-z 23107919

[B77] Hoang N. V. Furtado A. Botha F. C. Simmons B. A. Henry R. J. (2015a). Potential for genetic improvement of sugarcane as a source of biomass for biofuels. 3:182. 10.3389/fbioe.2015.00182 26636072 PMC4646955

[B78] Hoang N. V. Furtado A. Mason P. J. Marquardt A. Kasirajan L. Thirugnanasambandam P. P. (2017). A survey of the complex transcriptome from the highly polyploid sugarcane genome using full-length isoform sequencing and de novo assembly from short read sequencing. 18:395. 10.1186/s12864-017-3757-8 28532419 PMC5440902

[B79] Hoang N. V. Furtado A. Mcqualter R. B. Henry R. J. (2015b). Next generation sequencing of total DNA from sugarcane provides no evidence for chloroplast heteroplasmy. 1–2 33–45. 10.1016/j.neps.2015.10.001

[B80] Hotta C. Lembke C. Domingues D. Ochoa E. Cruz G. Q. Melotto-Passarin D. (2010). The biotechnology roadmap for sugarcane improvement. 3 75–87. 10.1007/s12042-010-9050-5

[B81] Irvine J. E. (1999). *Saccharum* species as horticultural classes. 98 186–194. 10.1007/s001220051057

[B82] International Rice Genome Sequencing Project (2005). The map-based sequence of the rice genome. 436 793–800. 10.1038/nature03895 16100779

[B83] Jackson S. Chen Z. J. (2010). Genomic and expression plasticity of polyploidy. 13 153–159. 10.1016/j.pbi.2009.11.004 20031477 PMC2880571

[B84] Jannoo N. Grivet L. Chantret N. Garsmeur O. Glaszmann J. C. Arruda P. (2007). Orthologous comparison in a gene-rich region among grasses reveals stability in the sugarcane polyploid genome. 50 574–585. 10.1111/j.1365-313X.2007.03082.x 17425713

[B85] Jia J. Zhao S. Kong X. Li Y. Zhao G. He W. (2013). Aegilops tauschii draft genome sequence reveals a gene repertoire for wheat adaptation. 496 91–95. 10.1038/nature12028 23535592

[B86] Jiao Y. Peluso P. Shi J. Liang T. Stitzer M. C. Wang B. (2017). Improved maize reference genome with single-molecule technologies. 546 524–527. 10.1038/nature22971 28605751 PMC7052699

[B87] Kim C. Lee T. -H. Compton R. O. Robertson J. S. Pierce G. J. Paterson A. H. (2013). A genome-wide BAC end-sequence survey of sugarcane elucidates genome composition, and identifies BACs covering much of the euchromatin. 81 139–147. 10.1007/s11103-012-9987-x 23161199

[B88] Kim C. Wang X. Lee T.-H. Jakob K. Lee G.-J. Paterson A. H. (2014). Comparative analysis of *Miscanthus* and *Saccharum* reveals a shared whole-genome duplication but different evolutionary fates. 26 2420–2429. 10.1105/tpc.114.125583 24963058 PMC4114942

[B89] Koren S. Phillippy A. M. (2015). One chromosome, one contig: complete microbial genomes from long-read sequencing and assembly. 23 110–120. 10.1016/j.mib.2014.11.014 25461581

[B90] Koren S. Schatz M. C. Walenz B. P. Martin J. Howard J. T. Ganapathy G. (2012). Hybrid error correction and de novo assembly of single-molecule sequencing reads. 30 693–700. 10.1038/nbt.2280 22750884 PMC3707490

[B91] Le Cunff L. Garsmeur O. Raboin L. M. Pauquet J. Telismart H. Selvi A. (2008). Diploid/polyploid syntenic shuttle mapping and haplotype-specific chromosome walking toward a rust resistance gene (Bru1) in highly polyploid sugarcane (2n ∼ 12x ∼ 115). 180 649–660. 10.1534/genetics.108.091355 18757946 PMC2535714

[B92] Lee H. Margarido G. R. A. Schatz M. Lembke C. Souza G. Heckerman D. (2015). “Sugarcane genome de novo assembly challenges,” in (San Diego, CA: PAG).

[B93] Lee S.-I. Kim N.-S. (2014). Transposable elements and genome size variations in plants. 12 87–97. 10.5808/GI.2014.12.3.87 25317107 PMC4196380

[B94] Levasseur A. Pontarotti P. (2011). The role of duplications in the evolution of genomes highlights the need for evolutionary-based approaches in comparative genomics. 6:11. 10.1186/1745-6150-6-11 21333002 PMC3052240

[B95] Li M. Liang Z. Zeng Y. Jing Y. Wu K. Liang J. (2016). De novo analysis of transcriptome reveals genes associated with leaf abscission in sugarcane (*Saccharum officinarum* L.). 17:195. 10.1186/s12864-016-2552-2 26946183 PMC4779555

[B96] Lin Y.-R. Draye X. Qian X. Ren S. Zhu L.-H. Tomkins J. (2000). Locus-specific contig assembly in highly-duplicated genomes, using the BAC-RF method. 28:E23. 10.1093/nar/28.7.e23 10710440 PMC102806

[B97] Ling H.-Q. Zhao S. Liu D. Wang J. Sun H. Zhang C. (2013). Draft genome of the wheat A-genome progenitor *Triticum urartu*. 496 87–90. 10.1038/nature11997 23535596

[B98] Lisch D. (2013). How important are transposons for plant evolution? 14 49–61. 10.1038/nrg3374 23247435

[B99] Loman N. J. Quick J. Simpson J. T. (2015). A complete bacterial genome assembled de novo using only nanopore sequencing data. 12 733–735. 10.1038/nmeth.3444 26076426

[B100] Mace E. S. Tai S. Gilding E. K. Li Y. Prentis P. J. Bian L. (2013). Whole-genome sequencing reveals untapped genetic potential in Africa’s indigenous cereal crop sorghum. 4:2320. 10.1038/ncomms3320 23982223 PMC3759062

[B101] Madoui M.-A. Engelen S. Cruaud C. Belser C. Bertrand L. Alberti A. (2015). Genome assembly using Nanopore-guided long and error-free DNA reads. 16:327. 10.1186/s12864-015-1519-z 25927464 PMC4460631

[B102] Manners J. M. (2011). “Functional genomics of sugarcane,” in Vol. 60 eds Kader J. C. Delseny M. (Cambridge, MA: Academic Press) 89–168.

[B103] Margarido G. R. A. Heckerman D. (2015). ConPADE: genome assembly ploidy estimation from next-generation sequencing data. 11:e1004229. 10.1371/journal.pcbi.1004229 25880203 PMC4400156

[B104] Matsuoka S. Kennedy A. J. dos Santos E. G. D. Tomazela A. L. Rubio L. C. S. (2014). Energy cane: its concept, development, characteristics, and prospects. 2014:597275 10.1155/2014/597275

[B105] McCormick R. F. Truong S. K. Sreedasyam A. Jenkins J. Shu S. Sims D. (2018). The *Sorghum bicolor* reference genome: improved assembly, gene annotations, a transcriptome atlas, and signatures of genome organization. 93 338–354. 10.1111/tpj.13781 29161754

[B106] McCoy R. C. Taylor R. W. Blauwkamp T. A. Kelley J. L. Kertesz M. Pushkarev D. (2014). Illumina TruSeq synthetic long-reads empower de novo assembly and resolve complex, highly-repetitive transposable elements. 9:e106689. 10.1371/journal.pone.0106689 25188499 PMC4154752

[B107] Metcalfe C. J. Oliveira S. G. Gaiarsa J. W. Aitken K. S. Carneiro M. S. Zatti F. (2015). Using quantitative PCR with retrotransposon-based insertion polymorphisms as markers in sugarcane. 66 4239–4250. 10.1093/jxb/erv283 26093024 PMC4493790

[B108] Miller J. R. Koren S. Sutton G. (2010). Assembly algorithms for next-generation sequencing data. 95 315–327. 10.1016/j.ygeno.2010.03.001 20211242 PMC2874646

[B109] Ming R. Liu S.-C. Lin Y.-R. Da Silva J. Wilson W. Braga D. (1998). Detailed alignment of Saccharum and Sorghum chromosomes: comparative organization of closely related diploid and polyploid genomes. 150 1663–1682. 9832541 10.1093/genetics/150.4.1663 PMC1460436

[B110] Ming R. Liu S.-C. Moore P. H. Irvine J. E. Paterson A. H. (2001). QTL analysis in a complex autopolyploid: genetic control of sugar content in sugarcane. 11 2075–2084. 10.1101/gr.198801 11731498 PMC311218

[B111] Montenegro J. D. Golicz A. A. Bayer P. E. Hurgobin B. Lee H. Chan C.-K. K. (2017). The pangenome of hexaploid bread wheat. 90 1007–1013. 10.1111/tpj.13515 28231383

[B112] Morrell P. L. Buckler E. S. Ross-Ibarra J. (2012). Crop genomics: advances and applications. 13 85–96. 10.1038/nrg3097 22207165

[B113] Myers E. W. Sutton G. G. Delcher A. L. Dew I. M. Fasulo D. P. Flanigan M. J. (2000). A whole-genome assembly of *Drosophila*. 287 2196–2204. 10.1126/science.287.5461.2196 10731133

[B114] Nair N. V. Selvi A. Sreenivasan T. V. Pushpalatha K. N. Mary S. (2006). Characterization of intergeneric hybrids of *Saccharum* using molecular markers. 53 163–169. 10.1007/s10722-004-1810-3

[B115] Okura V. Da Silva F. R. Da Silva M. J. Kudrna D. Ammiraju J. S. Talag J. (2012). A BAC library of the SP80-3280 sugarcane variety (*Saccharum* sp.) and its inferred microsynteny with the sorghum genome. 5:185. 10.1186/1756-0500-5-185 22524198 PMC3419638

[B116] Ouyang S. Zhu W. Hamilton J. Lin H. Campbell M. Childs K. (2007). The TIGR rice genome annotation resource: improvements and new features. 35 D883–D887. 10.1093/nar/gkl976 17145706 PMC1751532

[B117] PacBio (2016). Available at: http://www.pacb.com/smrt-science/smrt-sequencing/read-lengths/ [accessed August 20 2016].

[B118] Panje R. Babu C. (1960). Studies in *Saccharum spontaneum* distribution and geographical association of chromosome numbers. 25 152–172. 10.1508/cytologia.25.152

[B119] Paterson A. (2012). Berlin: Springer Science & Business Media.

[B120] Paterson A. Souza G. Sluys M. V. Ming R. D’hont A. Henry R. (2010). “Structural genomics and genome sequencing,” in Robert H. Chittaranjan K. (Boca Raton, FL: CRC Press) 149–165.

[B121] Paterson A. H. Bowers J. E. Bruggmann R. Dubchak I. Grimwood J. Gundlach H. (2009). The *Sorghum bicolor* genome and the diversification of grasses. 457 551–556. 10.1038/nature07723 19189423

[B122] Paterson A. H. Bowers J. E. Chapman B. A. (2004). Ancient polyploidization predating divergence of the cereals, and its consequences for comparative genomics. 101 9903–9908. 10.1073/pnas.0307901101 15161969 PMC470771

[B123] Paterson A. H. Lin Y. R. Li Z. Schertz K. F. Doebley J. F. Pinson S. R. (1995). Convergent domestication of cereal crops by independent mutations at corresponding genetic Loci. 269 1714–1718. 10.1126/science.269.5231.1714 17821643

[B124] Paterson A. H. Moore P. H. Tew T. L. (2013). “The gene pool of *Saccharum* species and their improvement,” in ed. Paterson A. H. (New York, NY: Springer) 43–71.

[B125] Paterson A. H. Wang X. Li J. Tang H. (2012). “Ancient and recent polyploidy in monocots,” in eds Soltis P. S. Soltis D. E. (Berlin: Springer) 93–108. 10.1007/978-3-642-31442-1_6

[B126] Piperidis G. D’hont A. Hogarth D. (2001). “Chromosome composition analysis of various *Saccharum* interspecific hybrids by genomic in situ hybridisation (GISH),” in Vol. 2 Brisbane, QLD 565–566.

[B127] Piperidis G. Piperidis N. D’hont A. (2010). Molecular cytogenetic investigation of chromosome composition and transmission in sugarcane. 284 65–73. 10.1007/s00438-010-0546-3 20532565

[B128] PlantGDB (2017). Available at: http://www.plantgdb.org/SbGDB/ [accessed June 8 2017].

[B129] Price H. J. Dillon S. L. Hodnett G. Rooney W. L. Ross L. Johnston J. S. (2005). Genome evolution in the genus *Sorghum* (Poaceae). 95 219–227. 10.1093/aob/mci015 15596469 PMC4246720

[B130] Raj P. Selvi A. Prathima P. Nair N. (2016). Analysis of genetic diversity of *Saccharum* complex using chloroplast microsatellite markers. 18 141–148. 10.1007/s12355-015-0382-1

[B131] Riaño-Pachón D. Mattiello L. (2017). Draft genome sequencing of the sugarcane hybrid SP80-3280. 6:861. 10.12688/f1000research.11859.2 28713559 PMC5499785

[B132] Riaño-Pachón D. M. Mattiello L. Prado Da Cruz L. (2016). Technical Report. Sao Paulo: Laboratório Nacional de Ciência e Tecnologia do Bioetanol.

[B133] Roach B. Daniels J. (1987). “A review of the origin and improvement of sugarcane,” in São Paulo 1–31.

[B134] Rossi M. Araujo P. G. Paulet F. Garsmeur O. Dias V. M. Chen H. (2003). Genomic distribution and characterization of EST-derived resistance gene analogs (RGAs) in sugarcane. 269 406–419. 10.1007/s00438-003-0849-8 12733061

[B135] Sandmann S. De Graaf A. O. Karimi M. Van Der Reijden B. A. Hellström-Lindberg E. Jansen J. H. (2017). Evaluating variant calling tools for non-matched next-generation sequencing data. 7:43169. 10.1038/srep43169 28233799 PMC5324109

[B136] Schmutz J. Cannon S. B. Schlueter J. Ma J. Mitros T. Nelson W. (2009). Genome sequence of the palaeopolyploid soybean. 463 178–183. 10.1038/nature08670 20075913

[B137] Schnable P. S. Ware D. Fulton R. S. Stein J. C. Wei F. S. Pasternak S. (2009). The B73 maize genome: complexity, diversity, and dynamics. 326 1112–1115. 10.1126/science.1178534 19965430

[B138] Scortecci K. C. Creste S. Calsa T. Jr. Xavier M. A. Landell M. G. Figueira A. (2012). “Challenges, opportunities and recent advances in sugarcane breeding,” in ed. Abdurakhmonov I. (Rijeka: InTech).

[B139] Selvi A. Nair N. V. Noyer J. L. Singh N. K. Balasundaram N. Bansal K. C. (2005). Genomic constitution and genetic relationship among the tropical and subtropical Indian sugarcane cultivars revealed by AFLP. 45 1750–1757. 10.2135/cropsci2004.0528

[B140] Shearman J. R. Sonthirod C. Naktang C. Pootakham W. Yoocha T. Sangsrakru D. (2016). The two chromosomes of the mitochondrial genome of a sugarcane cultivar: assembly and recombination analysis using long PacBio reads. 6:31533. 10.1038/srep31533 27530092 PMC4987617

[B141] Simmonds N. W. (ed.) (1976). “Sugarcane,” in (London: Longmans) 104–108.

[B142] Singh R. Mishra S. K. Singh S. P. Mishra N. Sharma M. (2010). Evaluation of microsatellite markers for genetic diversity analysis among sugarcane species and commercial hybrids. 4:116. 12834055

[B143] SoGI (2017). Available at: ftp://occams.dfci.harvard.edu/pub/bio/tgi/data/Saccharum_officinarum/ [accessed June 20 2017].

[B144] Song Q. Chen Z. J. (2015). Epigenetic and developmental regulation in plant polyploids. 24 101–109. 10.1016/j.pbi.2015.02.007 25765928 PMC4395545

[B145] Souza G. M. Berges H. Bocs S. Casu R. D’hont A. Ferreira J. E. (2011). The sugarcane genome challenge: strategies for sequencing a highly complex genome. 4 145–156. 10.1007/s12042-011-9079-0

[B146] Sreenivasan T. V. Ahloowalia B. S. Heinz D. J. (1987). “Chapter 5 - cytogenetics,” in ed. Don J. H. (Amsterdam: Elsevier) 211–253.

[B147] Steuernagel B. Taudien S. Gundlach H. Seidel M. Ariyadasa R. Schulte D. (2009). De novo 454 sequencing of barcoded BAC pools for comprehensive gene survey and genome analysis in the complex genome of barley. 10:547. 10.1186/1471-2164-10-547 19930547 PMC2784808

[B148] Stevens K. A. Wegrzyn J. Zimin A. Puiu D. Crepeau M. Cardeno C. (2016). Sequence of the sugar pine megagenome. 204 1613–1626. 10.1534/genetics.116.193227 27794028 PMC5161289

[B149] SUCEST-FUN Database (2015). Available at: http://sucest-fun.org [accessed May 01 2015].

[B150] SUGESI (2017). Available at: http:// cnrgv.toulouse.inra.fr/fr/Projets/Canne-a-sucre/The-Sugarcane-Genome-Sequencing-Initiative-SUGESI-Strategies-for-Sequencing-a-Highly-Complex-Genome [accessed May 25 2017].

[B151] Swaminathan K. Chae W. B. Mitros T. Varala K. Xie L. Barling A. (2012). A framework genetic map for *Miscanthus sinensis* from RNAseq-based markers shows recent tetraploidy. 13:142. 10.1186/1471-2164-13-142 22524439 PMC3355032

[B152] Swigoňová Z. Lai J. Ma J. Ramakrishna W. Llaca V. Bennetzen J. L. (2004). Close split of Sorghum and maize genome progenitors. 14 1916–1923. 10.1101/gr.2332504 15466289 PMC524415

[B153] Tambarussi E. V. Melotto-Passarin D. M. Gonzalez S. G. Brigati J. B. Jesus F. A. D. Barbosa A. L. (2009). In silico analysis of simple sequence repeats from chloroplast genomes of Solanaceae species. 9 344–352. 10.12702/1984-7033.v09n04a09 26739748

[B154] Tanaka T. Antonio B. A. Kikuchi S. Matsumoto T. Nagamura Y. Numa H. (2008). The rice annotation project database (RAP-DB): 2008 update. 36 D1028–D1033. 18089549 10.1093/nar/gkm978 PMC2238920

[B155] The Arabidopsis Genome Initiative (2000). Analysis of the genome sequence of the flowering plant *Arabidopsis thaliana*. 408 796–815. 10.1038/35048692 11130711

[B156] Tomkins J. P. Yu Y. Miller-Smith H. Frisch D. A. Woo S. S. Wing R. A. (1999). A bacterial artificial chromosome library for sugarcane. 99 419–424. 10.1007/s001220051252 22665173

[B157] Treangen T. J. Salzberg S. L. (2011). Repetitive DNA and next-generation sequencing: computational challenges and solutions. 13 36–46. 10.1038/nrg3117 22124482 PMC3324860

[B158] van Dijk E. L. Auger H. Jaszczyszyn Y. Thermes C. (2014). Ten years of next-generation sequencing technology. 30 418–426. 10.1016/j.tig.2014.07.001 25108476

[B159] Vettore A. L. Silva F. R. D. Kemper E. L. Arruda P. (2001). The libraries that made SUCEST. 24 1–7. 10.1590/S1415-47572001000100002

[B160] Vicentini R. Bottcher A. Brito MDos S. Dos Santos A. B. Creste S. Landell M. G. (2015). Large-scale transcriptome analysis of two sugarcane genotypes contrasting for lignin content. 10:e0134909. 10.1371/journal.pone.0134909 26241317 PMC4524650

[B161] Vidigal P. M. Coelho A. S. Novaes E. Barbosa M. H. Peternelli L. A. (2016). Complete chloroplast genome sequence and annotation of the *Saccharum* hybrid cultivar RB867515. 4:e01157-16. 10.1128/genomeA.01157-16 27738049 PMC5064122

[B162] Viola V. R. Lekshmi M. Premachandran M. (2011). Differentiation of cytoplasm of *Saccharum* and *Erianthus* species by mitochondrial DNA polymorphism. 71 1–3.

[B163] Wang J. Roe B. Macmil S. Yu Q. Murray J. E. Tang H. (2010). Microcollinearity between autopolyploid sugarcane and diploid sorghum genomes. 11:261. 10.1186/1471-2164-11-261 20416060 PMC2882929

[B164] Wang X. Shi X. Hao B. Ge S. Luo J. (2005). Duplication and DNA segmental loss in the rice genome: implications for diploidization. 165 937–946. 10.1111/j.1469-8137.2004.01293.x 15720704

[B165] Wendel J. F. (2015). The wondrous cycles of polyploidy in plants. 102 1753–1756. 10.3732/ajb.1500320 26451037

[B166] Yu J. Hu S. Wang J. Wong G. K. Li S. Liu B. (2002). A draft sequence of the rice genome (*Oryza sativa* L. ssp. *indica*). 296 79–92. 10.1126/science.1068037 11935017

[B167] Zhang J. S. Nagai C. Yu Q. Y. Pan Y. B. Ayala-Silva T. Schnell R. J. (2012). Genome size variation in three *Saccharum* species. 185 511–519. 10.1007/s10681-012-0664-6

[B168] Zhu J. Zhou H. Pan Y. Lu X. (2014). Genetic variability among the chloroplast genomes of sugarcane (*Saccharum* spp.) and its wild progenitor species *Saccharum spontaneum* L. 13 3037–3047. 10.4238/2014.January.24.3 24615073

